# Characterization and Comparison of the Divergent Metabolic Consequences of High-Sugar and High-Fat Diets in Male Wistar Rats

**DOI:** 10.3389/fphys.2022.904366

**Published:** 2022-07-04

**Authors:** Liske Kotzé-Hörstmann, Annibale Cois, Rabia Johnson, Lawrence Mabasa, Samukelisiwe Shabalala, Paul J. Van Jaarsveld, Hanél Sadie-Van Gijsen

**Affiliations:** ^1^ Non-Communicable Diseases Research Unit, South African Medical Research Council, Cape Town, South Africa; ^2^ Centre for Cardio-metabolic Research in Africa (CARMA), Division of Medical Physiology, Department of Biomedical Sciences, Faculty of Medicine and Health Sciences, Stellenbosch University, Stellenbosch, South Africa; ^3^ Division of Health Systems and Public Health, Department of Global Health, Faculty of Medicine and Health Sciences, Stellenbosch University, Stellenbosch, South Africa; ^4^ Division of Epidemiology and Biostatistics, School of Public Health and Family Medicine, University of Cape Town, Cape Town, South Africa; ^5^ Biomedical Research and Innovation Platform, South African Medical Research Council, Tygerberg, South Africa; ^6^ Department of Biochemistry and Microbiology, University of Zululand, KwaDlangezwa, South Africa

**Keywords:** obesity, rodent models, obesogenic diets, dietary intervention studies, metabolic fingerprinting, multivariate analysis

## Abstract

Diet-induced obesity (DIO) in laboratory rodents can serve as a model with which to study the pathophysiology of obesity, but obesogenic diets (high-sugar and/or high-fat) are often poorly characterised and simplistically aimed at inducing metabolic derangements for the purpose of testing the therapeutic capacity of natural products and other bioactive compounds. Consequently, our understanding of the divergent metabolic responses to different obesogenic diet formulations is limited. The aim of the present study was to characterise and compare differences in the metabolic responses induced by low-fat, medium-fat/high-sugar and high-fat diets in rats through multivariate statistical modelling. Young male Wistar rats were randomly assigned to CON (laboratory chow, low-fat), OB1 (high-sugar, medium-fat) or OB2 (high-fat) dietary groups (*n* = 24 each) for 17 weeks, after which metabolic responses were characterised. Projection-based multivariate analyses (principal component analysis (PCA) and orthogonal partial least squares-discriminant analysis (OPLS-DA)) were used to explore the associations between measures of body composition and metabolism. Furthermore, we conducted a systematic literature survey to examine reporting trends in rat dietary intervention studies, and to determine how the metabolic responses observed in the present study compared to other recently published studies. The OB1 and OB2 dietary regimens resulted in distinct metabolic profiles, with OB1 characterised by perturbations in insulin homeostasis and adipose tissue secretory function, while OB2 was characterised by altered lipid and liver metabolism. This work therefore confirms, by means of direct comparison, that differences in dietary composition have a profound impact on metabolic and pathophysiological outcomes in rodent models of DIO. However, through our literature survey we demonstrate that dietary composition is not reported in the majority of rat dietary intervention studies, suggesting that the impact of dietary composition is often not considered during study design or data interpretation. This hampers the usefulness of such studies to provide enhanced mechanistic insights into DIO, and also limits the translatability of such studies within the context of human obesity.

## Introduction

The global prevalence of obesity has more than tripled since 1975, and obesity in adults is now more prevalent than undernutrition. Approximately 40% of adults globally are overweight [body mass index (BMI) ≥ 25 kg/m^2^] while 13% of adults are obese (BMI ≥ 30 kg/m^2^) (World Obesity Federation, 2022). Obesity is associated with several co-morbidities including type II diabetes (T2D), various types of cancer and cardiovascular disease (Guh et al., 2009), as well as severe economic burden (Loeppke et al., 2009; Tremmel et al., 2017) and increased mortality (Whitlock et al., 2009).

The World Health Organisation (WHO) has set a global target to halt the rise in obesity by 2025, but recent data suggest that it is unlikely that this target will be achieved (Lobstein and Brindsen, 2020). A contributing factor to the lack of success at addressing human obesity may be our over-reliance on inadequately characterised rodent models of diet-induced obesity with which to study the mechanisms underpinning the establishment and progression of obesity. For clear ethical reasons, obesity cannot be induced in humans for research purposes, while longitudinal cohort studies, which aim to identify associations between genetic and lifestyle factors and the natural onset of obesity in humans, only yield results over decades. Consequently, human obesity is often studied retrospectively by comparing individuals with established obesity to their lean counterparts, and therefore the systemic and cellular mechanisms involved in the development and progression of obesity, especially diet-induced obesity, are only partially known. A large body of work has been performed examining the progression of diet-induced obesity (DIO) in rodent models, but the validity and applicability of these models in the context of human obesity are hampered by inadequate study design and a poor understanding of the unique or divergent metabolic consequences of different obesogenic diet formulations. In the majority of rodent studies where obesity is induced by dietary intervention, the overall aim of these studies is to demonstrate the efficacy of a purported weight-loss product or metabolic modulator, such as the anti-oxidant or anti-diabetic properties of natural botanical extracts. In such studies, the simplistic goal of the dietary intervention is to induce metabolic derangements, in order to demonstrate the effectiveness of the tested product at reversing or ameliorating these DIO effects. This often results in a “blunt-force-trauma” approach, where pathology is induced through various extreme diets, such as high-fructose (HFr) or high-cholesterol (HC) content, or a combination of high-fat (HF) and high-sugar (HS) content (HFHS), with little consideration for the real-life applicability of such diets or the relative contributions of different dietary constituents to the final metabolic profile. To our knowledge, only three rodent dietary intervention studies (Dourmashkin et al., 2005; Pranprawit et al., 2013; Matias et al., 2018) have compared the metabolic profiles resulting from different types of obesogenic diets [HF, HS, HFHS and medium fat (MF)], and although these studies have demonstrated differences in the metabolic outcomes between the various diets examined, several informative parameters such as serum insulin, leptin and adiponectin levels were not reported. As a result, much remains to be documented about the divergent metabolic consequences of obesogenic diets with different proportions of fat, sugars and cholesterol in rodent models of DIO.

In the present study, the primary aim was to compare the metabolic outcomes of three different diets [control (diet CON); HS/MF (diet OB1) and HF/HC (diet OB2)] in rats by using standard univariate statistical analyses as well as multivariate statistical modelling approaches, including principal component analysis (PCA) and orthogonal partial least squares-discriminant analysis (OPLS-DA) modelling. The variables (measures) considered here included body composition, organ weights, markers of glucose and insulin homeostasis, serum lipid profiles and serum cytokine/adipokine concentrations. These measures are routinely reported in rodent DIO studies in which single obesogenic diets are compared with control diets. To the best of our knowledge, this is the first time that this specific analytical approach has been used to directly compare the divergent effects on body composition, organ indices and metabolic markers of these feeding regimes commonly implemented in rodent models of DIO. As a secondary aim of our study, we conducted a literature survey to report some of the trends and assumptions around what constitutes a “successful” or “appropriate” response to obesogenic high-sugar and high-fat diets in rodent models.

## Materials and Methods

### Ethical Approval

Ethical approval was obtained from the Stellenbosch University Research Ethics Committee: Animal Care and Use (ethics clearance number ACU-2018-6786). All experiments involving animals were performed according to the ARRIVE guidelines for *in vivo* animal research. All animals were housed at the Animal Research Facility, Tygerberg campus, Stellenbosch University.

### Materials

For the dietary intervention study, standard laboratory rat chow pellets (Rodent Breeder Customized Laboratory Animal Diet) were purchased from LabChef Research Nutrition, Stellenbosch, South Africa. Sweetened condensed milk, cooking fat (solid vegetable oil brick, consisting predominantly of palm oil) and sucrose were purchased from local retail supermarkets. Fructose (#F0127), cholesterol (#C8503) and casein (#C7078) were purchased from Sigma-Aldrich (Germany). Sodium pentobarbitone (Eutha-naze) was from Bayer South Africa. Vacuette 5 ml serum separator tubes (SSTs) were from Greiner Bio-one. The Milliplex MAP rat cytokine/chemokine magnetic bead panel (RECYTMAG-65K, five-plex: leptin, tumour necrosis factor-alpha (TNF-α), interleukin (IL)-18, IL-6 and IL-1α), rat/mouse insulin ELISA kits (#EZRMI-13K) and rat adiponectin ELISA kits (#EZRADP-62K) were purchased from Merck South Africa.

### Preparation and Composition of the Experimental Diets

The preparation of the diets used in the present study was described previously (Obasa et al., 2021; Smit et al., 2022). Diet CON consisted of standard laboratory rat chow pellets; diet OB1 was prepared by mixing CON pellets with sweetened condensed milk, sucrose and a moderate amount of cooking fat, and diet OB2 was prepared by mixing CON pellets with large amounts of cooking fat, fructose, cholesterol and casein. A composite sample of each diet was analysed by a commercial food and nutritional testing analytical laboratory accredited by the South African National Accreditation System. The macronutrient composition for each diet is presented in Table 1. In terms of percentage energy (%E) from macronutrients, diet CON was low-fat (17 %E from fat) and consisted predominantly of protein (40 %E) and carbohydrates (43 %E; mostly starch with only 8 %E from sugars). Diet OB1 had a high carbohydrate content (49 %E from carbohydrates, 36 %E from sugars) and a moderate fat content (34 %E), while diet OB2 had a high fat content (68 %E) and very low carbohydrate content (15 %E from carbohydrates, 9 %E from sugars) (Table 1). As a consequence of the preparation method for diets OB1 and OB2, which involved the mixing of the additional dietary constituents with CON chow pellets, both diet OB1 and OB2 had increased moisture content and reduced protein and fibre content, compared to the CON diet, but the moisture, protein and fibre content was similar between OB1 and OB2 (Table 1). The mineral content (mg/kJ) diets OB1 and OB2 were considerably lower than diet CON, with diet OB2 having the lowest mineral content (Table 1).

**TABLE 1:** Macronutrient composition of the three experimental diets.

Variable (unit)	Dietary group		
	CON	OB1	OB2
Energy (kJ/100 g)	1225	968	1350
Moisture content (g/100 g)	10.4	42.2	41.1
Mineral (ash) content (g/100 g)	8.11	2.5	2.22
Mineral (ash) content (mg/kJ)	6.6	2.6	1.6
Carbohydrate			
Glycaemic carbohydrate (g/100 g)	27.4	27	11.5
Starch (g/100 g)	22.1	6.9	4.7
Total sugar (g/100 g)	5.2	20.1	6.7
Glucose (g/100 g)	0.1	0	0.2
Sucrose (g/100 g)	4.6	17.5	1.5
Fructose (g/100 g)	0.3	0.1	4.4
Other sugars (maltose, lactose, galactose, trehalose) (g/100 g)	0.2	2.5 (consisting solely of lactose)	0.7
Fat/Lipid			
Total fat (g/100 g)	5.04	8.80	24.09
%E from fat	17	34	68
Saturated fat (g/100 g)	1.28	5.26	14.3
Mono-unsaturated fat (g/100 g)	1.28	2.61	7.42
Poly-unsaturated fat (g/100 g)	2.48	0.94	2.38
Cholesterol (mg/100 g)	44	11	440
Protein			
Protein (g/100 g)	25.8	9	12.9
Protein (mg/kJ)	21	9.3	9.6
Fibre			
Fibre (g/100 g)	16.8	4.0	5.5
Fibre (mg/kJ)	13.7	4.1	4.1

CON, standard laboratory control chow diet; OB1, high-sucrose/medium-fat diet; OB2, high-fat/fructose/cholesterol diet; %E, % energy.

Published guidelines (Benevenga et al., 1995) state that laboratory rat diets should contain 15% protein (w/w) for growing rats. Diets OB1 and OB2 contained 9 and 12.9% (w/w) protein respectively, which at first glance would appear to indicate these diets to be protein deficient. However, the considerable 4-fold higher moisture content of diets OB1 and OB2, compared to the CON diet, affected the relative weight of the food; therefore a more accurate measure of the protein content would be mg protein/kJ. When expressed as such, diets OB1 and OB2 contained 9.3 and 9.6 mg protein/kJ, respectively, which is above the minimum protein content of 6.0–7.4 mg protein/kJ recommended for growing rats (Benevenga et al., 1995). It can therefore be concluded that diets OB1 and OB2 were not protein deficient, although the protein content (w/w) was between 2- to 2.9-fold lower than in the CON diet.

### Dietary Intervention Study

Weanling male Wistar rats (approximately 6 weeks of age, body weight 170–200 g) were randomly assigned into one of the three dietary groups. The rats were housed in groups of four per cage, maintained at 22°C under a 12 h/12 h day/night cycle, and had *ad libitum* access to food and water. Animals were maintained on their respective diets for 17 weeks. Food intake (grams) and water consumption (ml) were recorded daily for each cage, and body weight was recorded weekly for each animal.

### Sample Collection

After 16 weeks of dietary intervention (8 days before euthanasia), animals were fasted overnight and at 09:00 in the morning, a low dose (10 mg) of sodium pentobarbitone was administered *via* intraperitoneal injection to induce mild sedation. Animals were subsequently briefly anesthetized with 2% isoflurane gas mixture to collect 1 ml blood from the jugular vein into SSTs for serum analyses. A tail prick was also performed to collect blood for measuring fasting blood glucose (FBG) with a GlucoPlus™ glucometer. Once the isoflurane anaesthesia was reversed, a sucrose solution (1 mg/g body weight) was administered by oral gavage. Blood glucose levels were measured at 3, 5, 10, 15, 20, 25, 30, 45, 60, 90, and 120 min.

After 17 weeks of dietary intervention, the rats were weighed and euthanized in the non-fasted state by intraperitoneal injection with an overdose (160 mg/kg body weight) of sodium pentobarbitone, and subsequent exsanguination. All euthanizations were performed between 09:00 and 11:00, in order to avoid the impact of circadian cycles on metabolic variables. Blood was collected into SSTs and internal organs were harvested and weighed. The perirenal visceral fat from one flank was dissected as described previously (Sadie-Van Gijsen et al., 2020) and weighed as a surrogate marker for total visceral fat, as it was previously found that obesogenic diets affect the volume of this fat depot in rats (Pickavance et al., 1999; Du Toit et al., 2008). Sonographic measurements of perirenal fat have also been validated as a marker of total visceral fat in humans (Kawasaki et al., 2008; Grima et al., 2010). The pancreata and livers were also dissected and weighed. Organ indices were calculated as organ weight/body weight X 100.

### Blood Biochemistry

Blood samples collected into SSTs were allowed to clot on ice, and the serum was separated by centrifugation at 4°C for 10 min at 1800 × *g*. Serum aliquots were stored at −80°C until analysed. Quantification of serum insulin, adiponectin, ALT (alanine transaminase), AST (aspartate transaminase) and lipid profiles were all performed on serum samples collected in the fasted state (before the OGTT), while leptin, tumour necrosis factor-alpha (TNF-α), interleukin (IL)-18, IL-6 and IL-1α were quantified in serum collected in the non-fasted state at exsanguination. An insulin conversion factor of 1 µU/ml = 0.04 ng/ml was used to convert the mass-based measurement of insulin to active insulin units, as per the manufacturers’ instructions. The derived insulin concentration values (µU/ml) along with fasting blood glucose concentrations were used to calculate HOMA2-IR, HOMA2-%S (insulin sensitivity) and HOMA2-%B (pancreatic beta-cell function) (Wallace et al., 2004), utilising the University of Oxford Diabetes Trials Unit HOMA2 calculator software (online calculator available at https://www.dtu.ox.ac.uk/homacalculator/). Blood lipid profile analyses and quantification of serum ALT and AST were performed by Pathcare licensed pathology laboratories (Cape Town, South Africa). Leptin, TNF-α, IL-18, IL-6 and IL-1α were quantified with a Milliplex MAP rat cytokine/chemokine magnetic bead panel (RECYTMAG-65K), utilising a Bio-plex 200 automated immunoassay array system and Bio-Plex Manager 6.1 software (Bio-Rad Laboratories) for data acquisition.

### Statistical Analysis and Multivariate Modelling

Data sets for each variable per diet group were assessed for departures from normality (visual inspection and Shapiro-Wilk normality test) and the presence of outliers. SPSS version 27 (IBM Corporation, Armonk, NY) and GraphPad Prism version 5 (GraphPad Software, San Diego, CA) were used for univariate analyses. Repeated measures analysis of variance (ANOVA) was used to compare dietary group differences in food intake and energy intake (documented per cage) over weeks 6–10 (as a representative sample of the total study duration). Statistically significant differences in the distribution of metabolic variables between CON, OB1 and OB2 dietary groups were identified by one-way ANOVA followed by Tukey’s multiple comparisons *post-hoc* test. Where data exhibited non-normal distribution, a Kruskal–Wallis test followed by Dunn’s multiple comparison *post-hoc* test was used. Multivariate data analysis was performed in SIMCA version 15 (Umetrics Umeå, Sweden). Data structure and clustering were explored by unsupervised PCA. OPLS-DA, a supervised projection-based analysis, was performed to assess the relative contribution of the different metabolic variables to discriminate between the dietary groups (Bylesjö et al., 2006). OPLS-DA can successfully tolerate the multi-collinearity between metabolic variables that is often present in biological data sets containing many highly correlated variables, such as in the present study. Scatter and loading plots, and Variable Importance in Projection (VIP) score plots were constructed and interpreted to identify the metabolic variables most relevant for dietary group discrimination (Xi et al., 2014). The performance of the OPLS-DA model was assessed with k-fold cross-validation, by calculating the goodness of fit parameter R^2^ and the predictive ability parameter Q^2^. Permutation test was used to assess the robustness of the results and the possibility of overfitting (Triba et al., 2015). All variables were mean-centred and scaled to unit-variance before inclusion in the models. A *p*-value of ≤0.05 was considered to define statistical significance.

### Literature Survey

In addition to the dietary intervention study, we utilised a systematic search strategy to conduct a survey of dietary intervention studies in rats indexed on PubMed, using the following search terms: “rat” AND “adiponectin” AND “diet” AND “insulin”. The purpose of the literature survey was two-fold: 1) to establish to what extent the metabolic responses observed in the present study corresponded with findings from similar dietary studies reported elsewhere; and 2) to identify current trends in study design, preferred rat strains and reporting standards in rat dietary intervention studies. Studies published between 1 January 2016 and 30 June 2021 were included, but studies that used genetic models of obesity and/or diabetes, or where a diabetic or pre-diabetic state was induced with streptozotocin (STZ), were excluded, as well as studies involving surgery or pre-natal programming. Studies on high-cholesterol, high-fat, high-sugar (sucrose or fructose) or high-carbohydrate diets (or any combination thereof) were included, while studies on high-protein diets were excluded. Other exclusion criteria were as follows: studies where final body weight or changes in body weight were not reported; studies where serum insulin and serum adiponectin concentrations were not reported; studies where no dietary control group (standard laboratory chow or an equivalent) was included; articles where the full text was not available.

## Results

### Energy Intake, Primary Metabolic Endpoints and Univariate Analyses

Data for all the primary metabolic endpoints included in the present study are summarized as mean ± standard deviation (SD) by dietary group in Table 2. Further characterisation of the data distribution (sample size, range, median, 25th percentile and 75th percentile) is provided in Supplementary Table S1, while raw data obtained during the OGTT is provided in Supplementary Table S2. Between weeks 6 and 10 the average food consumption in g/cage/week was highest in the OB1 dietary group and lowest in the OB2 dietary group (Table 2). However, when food consumption was expressed as energy intake (kJ/cage/week), using the values in Table 1, the energy intake between the different dietary groups were similar (Table 2). Given that diets OB1 and OB2 had similar protein and fibre content when expressed as mg/kJ (Table 1), protein and fibre intake was therefore also similar between OB1 and OB2, although protein and fibre intake in OB1 and OB2 was lower than in CON, due to the lower protein and fibre content of diets OB1 and OB2 compared to the CON diet (Table 1). When the mineral intake per dietary group was calculated using the values in Table 1, it was found that mineral consumption was highest in dietary group CON and significantly lower in dietary groups OB1 and OB2. Mineral intake in dietary group OB2 was also significantly lower than in OB1 (Table 2). The amount of weekly food intake within the different dietary groups was constant over the 5-week period between weeks 6 and 10 (results not shown).

**TABLE 2:** Food and energy intake and metabolic characteristics (end-point analysis) for each dietary group.

**Variable (unit)	Dietary group			
	CON	OB1	OB2	*p*-value*
Food intake (g/cage/wk)	722.87 (82.68)^a^	890.50 (72.57)^b^	662.07 (49.32)^c^	a vs. b; a vs. c; b vs. c: *p* < 0.01
Energy intake (kJ/cage/wk)	8855.12 (1012.88)^a^	8620.04 (702.51)^a^	8937.90 (665.76)^a^	n.s
Mineral intake (g/cage/wk)	58.62 (6.71)^a^	22.26 (1.81)^b^	14.70 (1.09)^c^	a vs. b; a vs. c; b vs. c: *p* < 0.001
Final body weight (g)	378.26 (38.35)^a^	430.26 (45.08)^b^	410.83 (38.63)^b^	a vs. b: *p* < 0.001
Perirenal visceral fat one flank (g)	0.89 (0.30)^a^	1.56 (0.50)^b^	1.46 (0.52)^b^	a vs. b: *p* < 0.001
Visceral adiposity index (% of final body weight)	0.47 (0.14)^a^	0.72 (0.20)^b^	0.70 (0.21)^b^	a vs. b: *p* < 0.001
Liver weight (g)	12.03 (1.51)^a^	11.99 (1.42)^a^	15.50 (1.70)^b^	a vs. b: *p* < 0.001
Liver index (% of final body weight)	3.18 (0.21)^a^	2.81 (0.28)^b^	3.81 (0.44)^c^	a vs. b; a vs. c; b vs. c: *p* < 0.001
Pancreas weight (g)	0.63 (0.16)^a^	0.62 (0.15)^a^	0.43 (0.08)^b^	a vs. b: *p* < 0.001
Pancreas index (% of final body weight)	0.17 (0.04)^a^	0.14 (0.03)^a^	0.11 (0.02)^b^	a vs. b: *p* < 0.01
Fasting blood glucose (mmol/L)	5.50 (0.61)^a^	5.98 (0.86)^a^	5.94 (0.77)^a^	n.s
OGTT AUC (arbitrary units)	194.18 (54.60)^a^	248.90 (53.52)^a^	254.34 (129.25)^a^	n.s
Fasting insulin (ng/ml)	1.96 (1.16)^a^	4.11 (1.93)^b^	2.89 (1.48)^a^	a vs. b: *p* < 0.001
HOMA2-IR	6.62 (3.53)^a^	11.18 (3.52)^b^	9.08 (4.00)^a^	a vs. b: *p* < 0.001
HOMA2-%B	299.10 (110.61)^a^	418.13 (120.39)^b^	343.52 (145.05)^a^	a vs. b: *p* < 0.01
HOMA2-%S	21.24 (16.45)^a^	10.14 (4.40)^b^	14.62 (11.06)^a,b^	a vs. b: *p* < 0.01
Fasting adiponectin (ng/ml)	55.91 (18.39)^a^	88.89 (26.29)^b^	60.81 (22.42)^a^	a vs. b: *p* < 0.001
Leptin (ng/ml)	16.34 (5.65)^a^	27.32 (9.31)^b^	14.09 (7.49)^a^	a vs. b: *p* < 0.001
TNF-α (pg/ml)	0.70 (0.52)^a^	0.51 (0.51)^a,b^	0.51 (0.93)^b^	a vs. b: *p* < 0.05
IL-18 (pg/ml)	173.18 (77.36)^a^	186.23 (54.78)^a^	292.64 (139.80)^b^	a vs. b: *p* < 0.01
Fasting TAG (mmol/L)	0.67 (0.26)^a^	1.24 (0.35)^b^	1.27 (0.42)^b^	a vs. b: *p* < 0.001
Fasting total cholesterol (mmol/L)	1.84 (0.28)^a^	1.67 (0.25)^a^	2.95 (0.65)^b^	a vs. b: *p* < 0.001
Fasting HDL-C (mmol/L)	1.18 (0.22)^a^	1.01 (0.13)^b^	1.13 (0.18)^a,b^	a vs. b: *p* < 0.05
Fasting LDL-C (mmol/L)	0.02 (0.07)^a^	0.03 (0.15)^a^	1.07 (0.49)^b^	a vs. b: *p* < 0.001
ALT (IU/L)	38.22 (8.80)^a^	42.38 (14.24)^a^	145.09 (94.80)^b^	a vs. b: *p* < 0.001
AST (IU/L)	104.44 (18.41)^a^	95.29 (18.71)^a^	148.43 (57.55)^b^	a vs. b: *p* < 0.01

Different lower-case letters ^a^, ^b^, and ^c^ denote statistically significant differences.

*Test for difference between dietary groups: ANOVA and Tukey’s post-hoc test (normally distributed variables) or Kruskal-Wallis and Dunn’s test (non-normal distribution).

**All data are shown as mean (± standard deviation), regardless of distribution. Additional descriptive statistics, including sample size for each analysis (N), median and interquartile range, can be found in Supplementary Table S1.

CON, standard laboratory control chow diet; OB1, high-sucrose/medium-fat diet; OB2, high-fat/fructose/cholesterol diet; ALT, alanine transaminase; AST, aspartate aminotransferase; HDL, high-density lipoprotein; HOMA, homeostatic model assessment; LDL, low-density lipoprotein; OGTT AUC, oral glucose tolerance test area under the curve; TAG, triacylglycerol; TNF-α, tumour necrosis factor-alpha; wk, week; n.s, no significant difference.

The final mean body weight of the rats in OB1 and OB2 were similar and significantly higher compared to the rats in CON (Table 2). Likewise, the mean perirenal fat mass (as a surrogate marker of total visceral fat) and the mean visceral adiposity index (VAI) were similar in OB1 and OB2 and significantly higher compared to CON. The mean liver weight and liver index were elevated in OB2 compared to CON and OB1, while the mean liver index was reduced in OB1 compared to CON and OB2. The mean pancreas weight and pancreas index were reduced in OB2 compared to CON and OB1. The mean OGTT AUC values and FBG concentrations were not significantly different between the three dietary groups. The mean fasting insulin concentrations and the HOMA2-IR index were significantly elevated in OB1 compared to CON and OB2. In OB1 the mean HOMA2-%S index (insulin sensitivity) was reduced and the mean HOMA2-%B index (pancreatic beta-cell function) was elevated compared to CON and OB2 (Table 2).

The mean serum triacylglycerol (TAG) concentration was elevated to the same extent in both OB1 and OB2, compared to CON. Mean total cholesterol and LDL-cholesterol concentrations were higher in OB2 than in CON and OB1, while the mean HDL-cholesterol concentration was slightly lower in OB1 than in CON and OB2. Mean serum ALT and AST concentrations were higher in OB2 compared to CON and OB1. Mean serum adiponectin and leptin concentrations were highest in OB1, with no differences between CON and OB2 (Table 2). Serum TNF-α concentrations were overall very low, and were below the minimum detectable concentration of the assay (<1.9 pg/ml) in many samples. For samples where TNF-α concentrations of <1.9 pg/ml were reported, these concentrations were estimated by the Bio-plex Manager software, extrapolating between the lowest assay standard and the negative control. Although mean TNF-α levels (Table 2) did not differ between OB1 and OB2, median TNF-α levels (Supplementary Table S1) were lowest in OB2 and significantly different from CON. Mean serum IL-18 concentrations were higher in OB2 compared to CON and OB1. IL-6 and IL-1α were undetectably low in all three dietary groups (<30.7 pg/ml for IL-6 and <4.2 pg/ml for IL-1α, according to the manufacturer’s information on the minimum detectable concentration for each analyte). For IL-6, this is consistent with other studies using young male Wistar rats, where reported serum IL-6 concentrations ranged between 1 and 19 pg/ml (Zhao et al., 2017; Peng et al., 2018; Vidé et al., 2018), and indicates that the assay utilised here may not be the most appropriate choice for the quantification of low IL-6 levels in young male Wistar rats.

### Metabolic Fingerprinting of High-Sugar and High-Fat Diet Responses Compared to Control Diet

**FIGURE 1:**
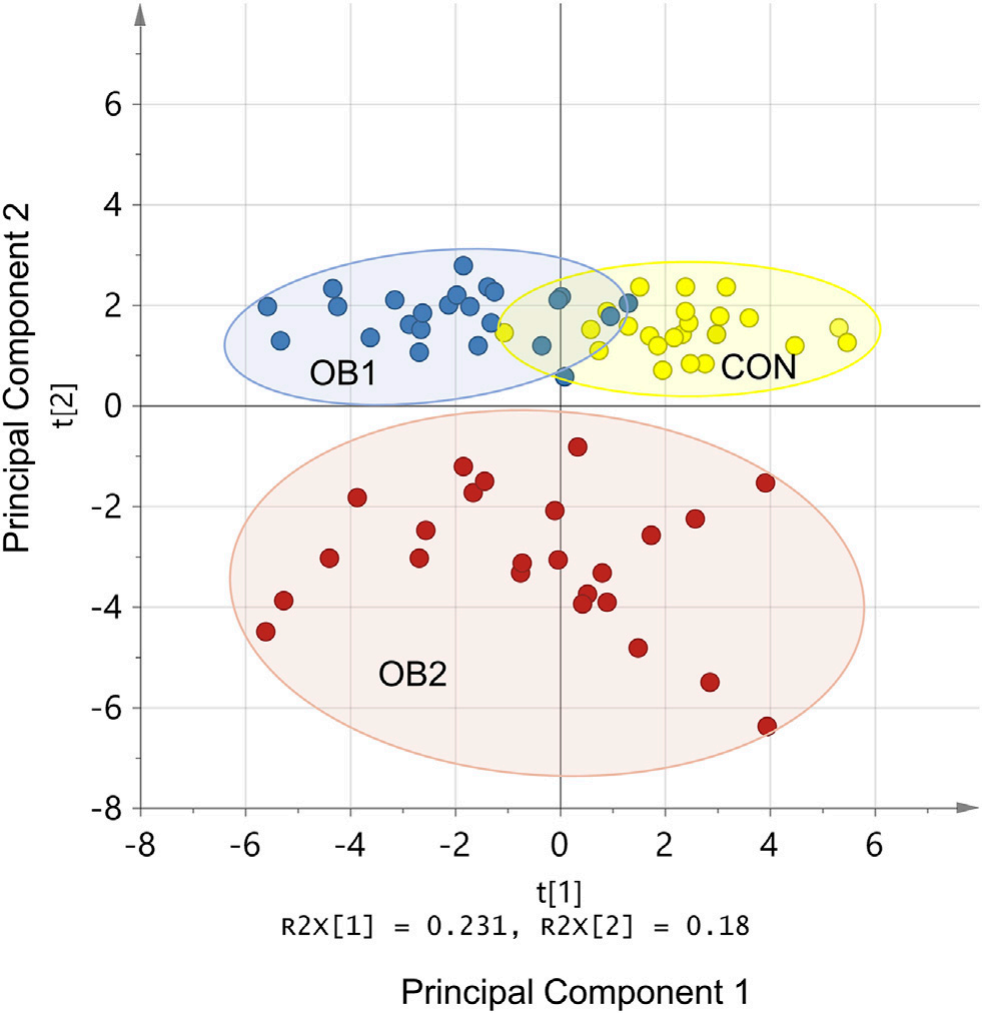
PCA model standardised scores plot. R2x[1] and R2x[2] indicate the proportion of variance explained by the first and second principal components, respectively. Only the first and second principal components are shown. The coloured dots (individual rats) indicate dietary group membership (CON = yellow, OB1 = blue, and OB2 = red) and the ellipses indicate the 95% confidence interval for each data set.

Principal component analysis (PCA) is a tool for reducing the number of variables included in a statistical model, by identifying subsets of variables (principal components) that can effectively distinguish between experimental groups. When data on all the measured variables for the 72 rats were included in the PCA, the first two principal components accounted for 41% of the original variation, with component 1 accounting for 23% and component 2 accounting for 18% (*p* < 0.05 for both components) (Figure 1). Component 2 (of which the most influential variables were LDL-C, total cholesterol, ALT, AST, liver weight and pancreas weight) achieved unambiguous separation between OB2 and non-OB2 animals, while component 1 (of which the most influential variables were OGTT AUC, blood glucose concentrations at the 20, 30 and 60 min time-points of the OGTT, visceral fat weight and visceral adiposity index) distinguished between CON and OB1, with very little overlap between these two dietary groups (Figure 1). The clear separation of the dataset into three distinguishable sub-groups in the PCA score plot (Figure 1), which corresponded to a large extent with the three dietary groups, indicated that a substantial share of the observed variation in the metabolic variables could be ascribed to inter-group differences rather than heterogeneity within the dietary groups.

**FIGURE 2:**
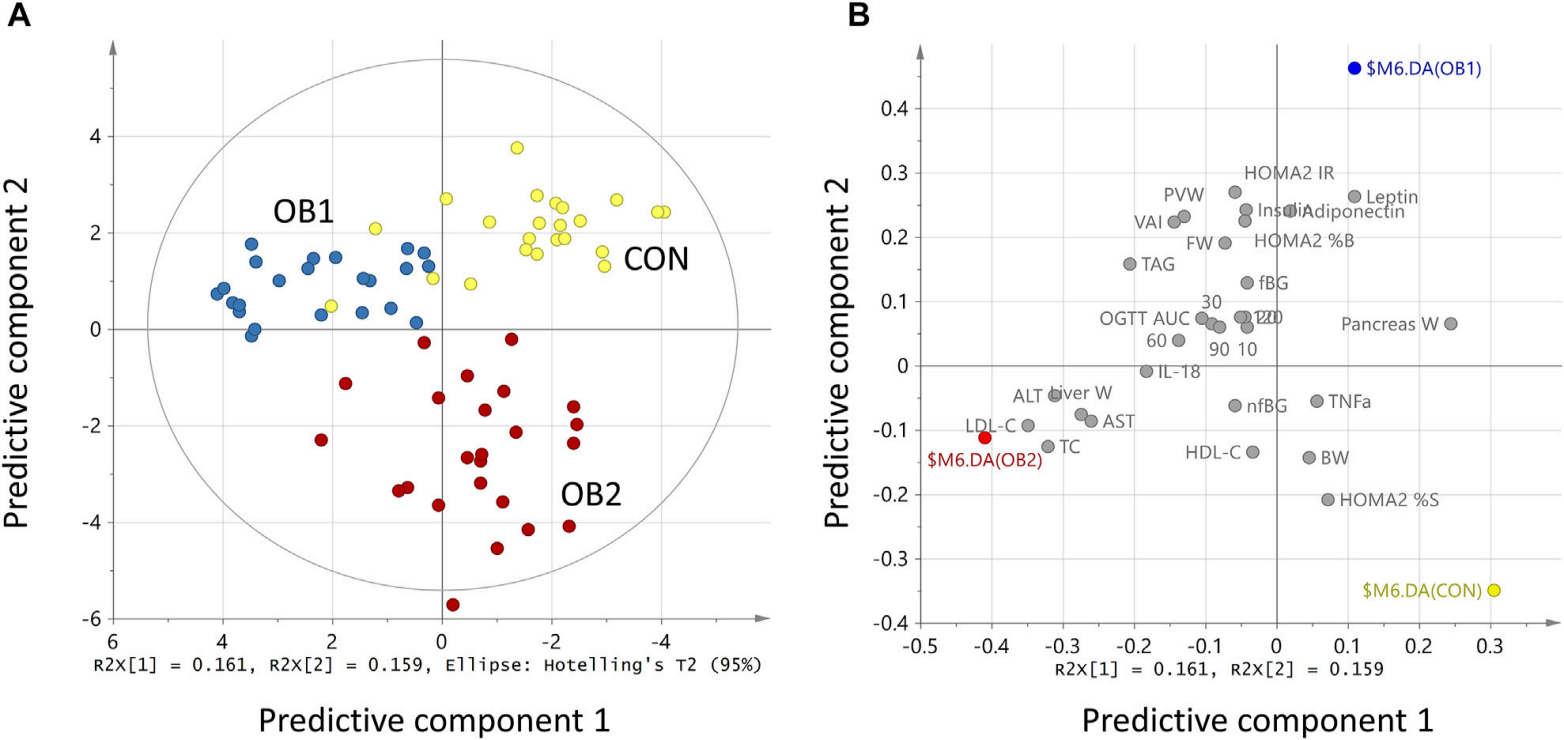
OPLS-DA model scores scatter plot **(A)** and loadings scatter plot **(B)**. R2x[1] and R2x[2] indicate the proportion of the variance explained by the first and second predictive component, respectively. In **(A)**, the coloured dots (individual rats) indicate dietary group membership, with CON = yellow, OB1 = blue and OB2 = red. In **(B)**, dietary groups are represented by coloured dots (CON = yellow, OB1 = blue, and OB2 = red) and the metabolic variables by grey dots. Values were scaled as proportion to R2X **(A)** and normalised to unit length **(B)**.

**FIGURE 3:**
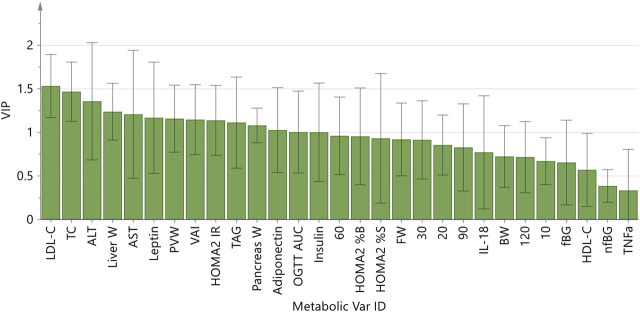
OPLS-DA model Variable Importance for Projection (VIP) scores plot.

**FIGURE 4:**
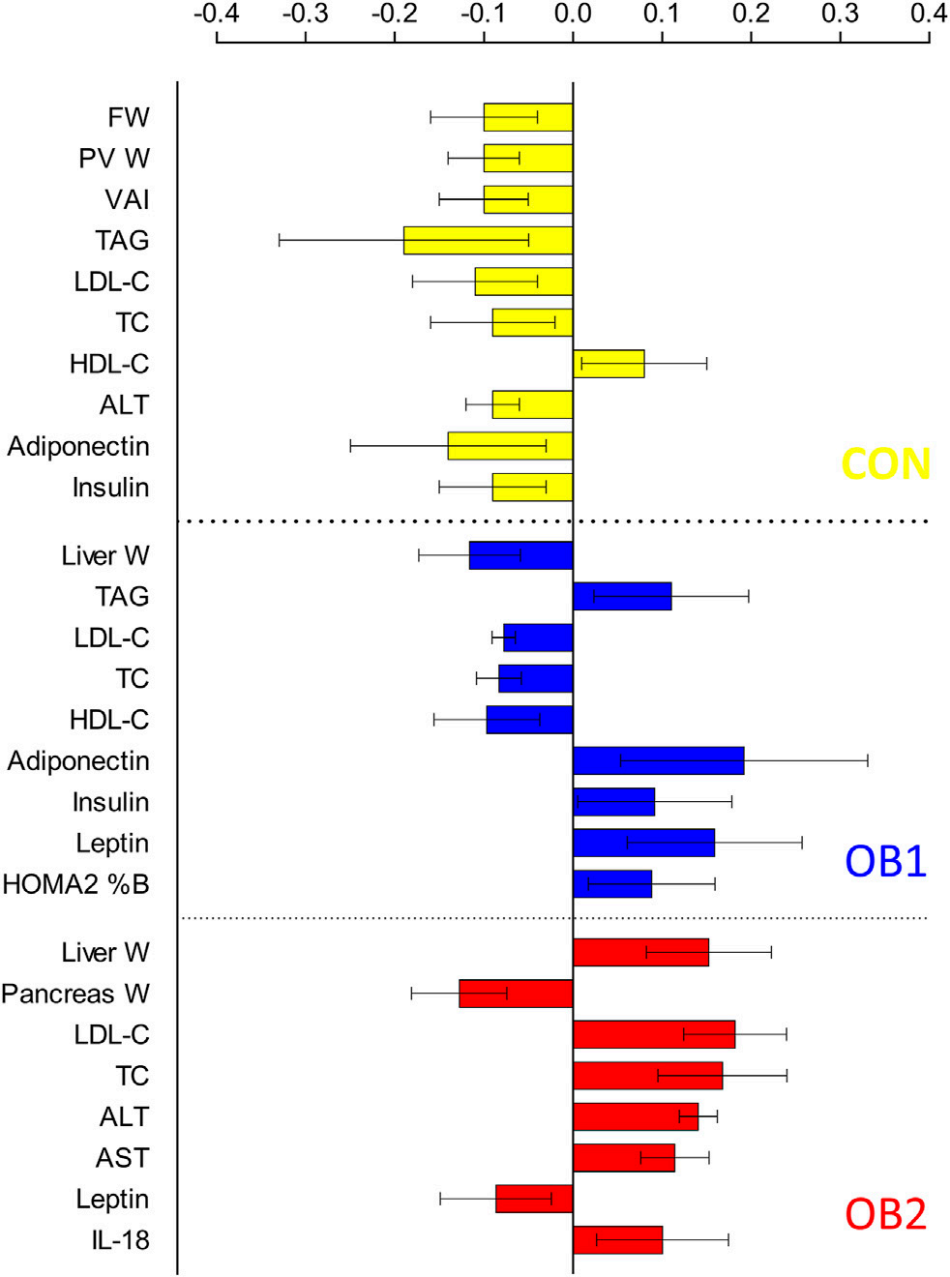
Multivariate association of metabolic variables with dietary group: partial correlation coefficients.

The strong performance of the PCA enabled us to construct an OPLS-DA model with low risk of spurious or aggressively forced group separation (Worley and Powers, 2016), to determine which metabolic variables most significantly contributed to the discrimination between the dietary groups. OPLS-DA model outputs are summarised in the scores and loadings plots (Figures 2A,B), and the VIP scores plot (Figure 3), while the model parameters are summarised in Supplementary Table S3. Significant group separation was achieved along the first two predictive components (R2X = 0.31) and one orthogonal component (R2X = 0.172) (Figure 2A). The second predictive component of the OPLS-DA model sufficiently separated OB2 from OB1 and CON (based on liver and lipid metabolism measures, as well as pancreas weight), whereas predictive component one separated CON and OB1 dietary groups (based mainly on insulin and glucose measures) (Figure 2A). The VIP score plot in Figure 3 indicates that markers of liver and lipid metabolism were the strongest predictors (highest VIP scores) of dietary group membership, followed by measures of visceral adiposity and insulin resistance. In this model, glucose measures derived from the OGTT (FBG, glucose levels at individual time-points and AUC) and inflammatory markers (TNF-α and IL-18) provided information less relevant to dietary group membership. The pattern of association of the metabolic markers with the CON, OB1 and OB2 dietary groups are presented in Figure 4. Overall, CON and OB1 dietary groups were associated with more favourable lipid (lower TAG, total cholesterol and LDL-C) and liver (lower ALT and liver weight) metabolism profiles compared to the OB2 dietary group, which associated with higher liver weight, ALT and AST concentrations. CON was associated with higher HDL-cholesterol compared to OB1, while OB1 was associated with higher serum insulin, adiponectin and leptin levels, compared to CON.

The validation of the OPLS-DA model provided satisfactory results. The values of the fit parameter R^2^ = 0.713 and the predictive ability parameter Q^2^ = 0.663 were similar and well above the cut-off of 0.5 commonly used in literature as an indication of good predictive power (Triba et al., 2015). The area under the receiver operating characteristic (ROC) curve indicated high discriminatory power with values close to the unity for the three dietary groups (Supplementary Figure S1). The negative intercepts of the Q^2^ regression lines in the permutation plots (Supplementary Figures S2–S4) and the minimal overlapping of the original and permuted points (7/500, 9/500 and 6/500 overlapping points for diets CON, OB1, OB2 respectively) supports the validity of the model and do not suggest overfitting (SIMCA^®^ 15 User Guide, www.sartorius.com).

### Outcomes of the Literature Survey

The purpose of the literature survey was to compare our study to previous studies of similar design, in terms of the metabolic variables included and the impact of different dietary interventions on these variables. The search terms were chosen to select studies where comprehensive metabolic characterisations were performed, ensuring sufficient comparability with our present study design and observations. The raw data of the literature survey are provided in Supplementary Table S4, with references listed in Supplementary Datasheet S1; a brief summary is presented here.

The final survey included 72 publications with 77 comparisons between obesogenic diets and control diets; 4 publications (Marques et al., 2016; Sangüesa et al., 2017; Moreno-Fernández et al., 2018; Muhammad et al., 2021) included more than one comparison. Diet formulations were not standardised and most obesogenic diets were used only once across the 72 publications, except where the same diet was used in multiple studies by the same research group, or where commercially available research diets were used. Male Wistar rats were the model of choice (39/77), followed by male Sprague-Dawley rats (29/77). Female rats were only used in 9 (4/9 Wistar; 5/9 Sprague-Dawley) of the 77 comparisons. Young rats, defined as either ≤200 g body weight or ≤8 weeks old, were used in more than half of the comparisons (47/77). The majority of comparisons (62/77) induced obesity in order to test a product or intervention for its capacity to reverse the diet-induced metabolic derangements. Two studies (Sangüesa et al., 2017; Moreno-Fernández et al., 2018) compared the effects of different sugars (fructose vs. glucose), but none of the included studies compared the effects of different types of fat in obesogenic diets. Only one study compared the impact of the diets between males and females (Muhammad et al., 2021), and only one study compared the effects between different rat strains (male Wistar vs. Sprague-Dawley rats) (Marques et al., 2016). None of the included studies compared the effects of obesogenic diets between young and old rats. Perhaps the most disappointing finding was that fewer than 50% of the included comparisons (34/77) provided the complete macronutrient composition and energy content (kJ/g or kcal/g) of the different diets used, and 49/77 did not report the total energy intake in kJ or kcal of the different dietary groups. Many of the publications included in the survey supplied incomplete lists of the dietary constituents, for example, dietary ingredients such as the mass of lard or soybean oil expressed as g/100 g food, but this information was not sufficient to allow for macronutrient comparisons between studies. A large proportion of the comparisons (60/77) stated that a high-fat diet was utilised (high-fat, high-fat-high-sugar or high-fat-high-fructose), but in 36 of these 60 comparisons the fat content of the diet was either lower than that of commercially available validated high-fat diets for laboratory rodent research (≥40% energy from fat)^1^, or not enough information was provided to verify the fat content of the diet. Isocaloric energy intake between the control and obesogenic dietary groups was reported in only 7 of the 77 dietary comparisons.

A variety of metabolic responses to the obesogenic diets were reported, which showed a high degree of correspondence with the results of the present study (summarised in Table 3). Final body weight was higher compared to that of control rats in the majority of studies (62/77), regardless of dietary composition. However, in 4/7 studies where energy intake was isocaloric between the control and obesogenic dietary groups, the final body weights were similar between dietary groups. Where it was measured (53/77 comparisons), visceral adiposity was almost universally (50/53 comparisons) increased with obesogenic feeding. Fasting glucose concentration was elevated in 47/73 comparisons (not reported in four comparisons), and fasting insulin concentration was elevated in 60/77 comparisons. Where reported, the serum triacylglycerol concentration was elevated in 51/66 comparisons, and serum total cholesterol concentrations were elevated in 48/68 comparisons. Serum adiponectin concentration was elevated in 8/77 comparisons, decreased in 49/77 comparisons, and similar to the control dietary group in 20/77 comparisons. Serum leptin concentration was elevated in 43/52 comparisons where it was reported, and in 26/52 comparisons where both leptin and adiponectin concentrations were reported, an inverse correlation between adiponectin and leptin concentrations were found (lower adiponectin and higher leptin concentrations in the obesogenic dietary groups, compared to control groups).

**TABLE 3:** Summary of results of literature survey and comparison with present findings.

	Literature survey		
Variable	Number of comparisons where measured	Outcome in obesogenic dietary group, compared to CON	Present study (outcome in OB1 and OB2, compared to CON)
Final body weight	77/77 (Isocaloric: 7/7)	Higher in 62/77 (Isocaloric: Higher in 3/7)	Higher in OB1 and OB2
Visceral adiposity	53/77 (Isocaloric: 7/7)	Higher in 50/53 (Isocaloric: Higher in 6/7)	Higher in OB1 and OB2
Fasting blood glucose	73/77 (Isocaloric: 6/7)	Higher in 47/73 (Isocaloric: Higher in 3/6) No difference in 26/73 (Isocaloric: no difference in 3/6)	No difference between CON, OB1 and OB2
Fasting serum insulin	77/77 (Isocaloric: 7/7)	Higher in 60/77 (Isocaloric: Higher in 4/7)	Higher in OB1, no difference between CON and OB2
Serum triacylglycerol	66/77 (Isocaloric: 5/7)	Higher in 51/66 (Isocaloric: Higher in 3/5)	Higher in OB1 and OB2
Serum total cholesterol	68/77 (Isocaloric: 5/7)	Higher in 48/68 (Isocaloric: Higher in 1/5)	Higher in OB2, no difference between CON and OB1
Serum adiponectin	77/77 (Isocaloric: 7/7)	Higher in 8/77 Lower in 49/77 Not different in 20/77 (Isocaloric: Higher in 1/7 Lower in 4/7 Not different in 2/7)	Higher in OB1, no difference between CON and OB2
Serum leptin	52/77 (Isocaloric: 6/7)	Higher in 43/52 (Isocaloric: Higher in 4/6)	Higher in OB1, no difference between CON and OB2

CON, standard laboratory control chow diet; OB1, high-sucrose/medium-fat diet; OB2, high-fat/fructose/cholesterol diet. “Isocaloric” refers to the subset of 7 comparisons where energy intake did not differ between the control and obesogenic dietary groups. Additional information on the full set of studies included in the final survey can be found in Supplementary Table S4.

## Discussion

To our knowledge, this is the first study to compare the metabolic consequences in rats that were fed obesogenic diets containing different proportions of fat, cholesterol and sugars, using comprehensive metabolic analyses and multivariate statistical modelling. The univariate analyses demonstrated that increased visceral adiposity and hypertriglyceridemia were the only metabolic consequences common to both the OB1 and OB2 diets. In support of this observation, the applied OPLS-DA model demonstrated that there was very little overlap in the metabolic profiles between the three dietary groups. Variables related to insulin and adipose tissue metabolism featured more strongly in dietary group OB1 (high-sugar/medium-fat), while variables related to liver function and cholesterol metabolism were more prominently impacted in dietary group OB2 (high-fat/high-cholesterol). In particular, our observations that serum leptin and adiponectin concentrations were specifically increased only in response to the OB1 diet demonstrates that, perhaps counter-intuitively, adipose tissue function and metabolism are affected more profoundly by high-sugar feeding than high-fat feeding. In contrast, the high-fat/high-cholesterol diet specifically impacted liver function. In addition, while increased proportions of fat and sugar both resulted in increased serum triacylglycerol, increased serum cholesterol was only observed as a consequence of increased dietary cholesterol. Furthermore, although serum TNF-α and IL-6 concentrations were uninformative in our study, the increase in serum IL-18 concentration in dietary group OB2 nevertheless suggests a heightened systemic inflammatory state specific to the OB2 diet.

While the OB1 and OB2 obesogenic diets had lower fibre and protein content and higher moisture content than the control diet CON, the moisture, fibre and protein content of diets OB1 and OB2 was virtually identical. The increased moisture content and resultant enhanced palatability of diets OB1 and OB2, together with unrestricted access to the food, may potentially have stimulated hyperphagia, but this was apparently not the case in the present study, as the energy (kJ) intake was similar across all three dietary groups, irrespective of dietary composition. Therefore, it is unlikely that these factors (moisture, protein and fibre content, and energy intake) could have contributed to differences in the metabolic outcomes between the OB1 and OB2 dietary groups, although the lower fibre and protein content of diets OB1 and OB2 may have contributed to the differences between CON and OB1, or between CON and OB2 (Pezeshki et al., 2016; Pellizon and Ricci, 2018). This supports the conclusion that the divergent metabolic consequences of diets OB1 and OB2 are attributable solely to differences in the relative proportions of fat, cholesterol and sugar components of the diets. Mineral intake was lower in dietary groups OB1 and OB2, compared to CON, and lower in dietary group OB2, compared to OB1, which may have contributed to differences in metabolic outcomes between the three dietary groups. However, given that mineral intake is often not reported in rat DIO studies, as demonstrated through our literature survey where only 20 out of 77 included comparisons documented mineral intake, the metabolic impact of different levels of mineral intake against the background of different obesogenic diets remains largely uncharacterised.

The results of the present study correspond well with the findings of many previous studies, as determined by our literature survey. We observed very slight differences in the final body weights of the rats between the three different dietary groups. This is in agreement with the findings of the literature survey, which highlighted that obesogenic diets did not always result in increased body weight of the rats, compared to rats on the control diets, especially where energy intake was similar between dietary groups. Body weight was correspondingly also a relatively unimportant variable in the OPLS-DA model. In contrast, rats in both the OB1 and OB2 dietary groups exhibited increased visceral adiposity, it was a more important variable in the OPLS-DA model and it was increased in response to obesogenic feeding in almost all of the studies included in the literature survey irrespective of dietary composition. In addition, our finding of hypertriglyceridemia in response to both the OB1 and OB2 diets is in agreement with the majority of studies included in the literature survey, irrespective of dietary composition. However, while many comparisons included in the literature survey reported increased serum cholesterol concentrations in response to obesogenic feeding, our present study results demonstrate that this effect is influenced by dietary composition.

It is noteworthy that neither diet OB1 nor diet OB2 resulted in increased fasting blood glucose concentrations, but similar observations were made in approximately one-third of the comparisons included in our literature survey. We speculate that the absence of changes in fasting blood glucose may be related to other variables that also remained unchanged between the groups or only showed very small changes, such as calorie intake or final body weight. Alternatively, it is possible that different glucose-regulating mechanisms were involved in maintaining normoglycemia in OB1 and OB2, respectively. In contrast to most previous DIO studies where serum adiponectin concentrations were downregulated, we observed that serum adiponectin was upregulated in specifically the OB1 dietary group. In our literature survey, the serum adiponectin concentration was elevated in only 8 out of 77 studies, but notably all of these studies used diets with high-sugar (glucose, sucrose or fructose) content, similar to the OB1 diet in our study. In 7 of these 8 studies, the serum fasting insulin concentration was concomitantly elevated, as was observed in the present study in the OB1 dietary group. We speculate that this may be an adaptive response to the increased sugar load in these diets, whereby insulin-dependent glucose uptake capacity can be supported and enhanced by adiponectin-stimulated insulin-independent glucose uptake via the AMPK pathway (Rana et al., 2015), in an attempt to maintain normoglycemia. In 3 of these 8 studies, the fasting blood glucose concentrations were not increased in response to high-sugar feeding, and in the present study the fasting blood glucose concentrations were only elevated in five rats in the OB1 dietary group. Taken together, these observations suggest that this metabolic compensation strategy might be at least partially successful to maintain normoglycemia in the presence of sustained high-sugar feeding. In contrast, such a compensatory mechanism was clearly absent in rats on the OB2 diet, with serum insulin and adiponectin levels not significantly different to that of the CON dietary group. However, such a mechanism was likely also not required, given the very low sugar load of diet OB2. Our results also demonstrate that DIO-induced increases in serum triacylglycerol concentration are not always coupled with a downregulation of serum adiponectin concentration, given that the hypertriglyceridemia in dietary groups OB1 and OB2 occurred against the background of both elevated serum adiponectin (OB1 vs. CON) or unchanged adiponectin levels (OB2 vs. CON). These observations are also supported by the findings of our literature survey (Supplementary Table S4) and suggest that these changes in key metabolic variables are governed by a complex interplay of physiological mechanisms which still need to be elucidated.

Multivariate data analyses, such as OPLS-DA and PLS-DA, have gained popularity in metabolic research as a tool to integrate large numbers of metabolic variables into coherent statistical models that can explain overall outcomes, instead of isolated analyses that can only provide limited insights. Through the use of VIP plots, such modelling approaches can also assist in identifying variables of higher pathophysiological, diagnostic or therapeutic importance than conventionally used variables, and may contribute towards biomarker discovery. Several studies have utilised these modelling approaches to compare the metabolic impact of single obesogenic diets to that of control diets (Cai et al., 2021) and to comprehensively characterise the effects of traditional Chinese medicine products and other natural products against a background of high-fat feeding (Gooda Sahib Jambocus et al., 2016; Feng et al., 2018; Shi et al., 2020; Wang et al., 2020). Of relevance to the present study are other published works where multivariate data analyses have been used to improve our understanding of the differences between various rodent metabolic models, for example, by characterising the different metabolic profiles of lean, high-fat diet (HFD)-fed and HFD/streptozotocin (STZ)-induced T2D rats (Chen et al., 2021) or of HFD/STZ-induced T2D vs. STZ-induced type I diabetic rats (Mediani et al., 2018). The impact of diabetic status on the metabolic response to HFD has also been explored using this methodology (Lee et al., 2021). In addition, Letsinger et al. (2020) demonstrated how a high-fat/high-sugar diet differentially affects the gut metabolome between male and female mice, which is of particular importance given the predominance of male rodent use in animal dietary studies and the lack of studies comparing the responses of male and female rodents to obesogenic feeding, as identified in our literature survey. However, to our knowledge, our study is the first to directly compare the metabolic consequences in rats consuming three different diets, using multivariate data analyses.

In conclusion, our analyses clearly showed divergent metabolic consequences in rats resulting from obesogenic diets with different proportions of cholesterol, fat and sugar. Therefore, it can be concluded that dietary composition is a dominant contributor to the metabolic outcomes of these type of studies. This has profound implications for animal DIO studies, in particular where therapeutic or nutraceutical compounds are being tested against the background of such diets, as the metabolic impact of the obesogenic diet may influence the efficacy of the compound being tested. However, our literature survey demonstrated the vast heterogeneity of diets used under the “umbrella term” DIO, which hampers our ability to compare findings between diets, or to develop a comprehensive understanding of the metabolic consequences of any given type of obesogenic diet. Moreover, the lack of DIO studies performed in female animals, and the lack of direct comparisons between males and females means that we have very little insight into the impact of biological sex on the metabolic outcomes of such diets. This shortcoming needs to be urgently addressed by including female animals in the design of DIO studies, especially given that obesity is more prevalent among women than men (Lobstein et al., 2022). Taken together, our OPLS-DA model and our literature survey also confirm that there is no single “appropriate” or “successful” response to obesogenic feeding in rodents, but rather that the response to individual obesogenic diets will be dependent on dietary composition and will need to be characterised objectively in each study. Furthermore, excess nutrient supply results in oxidative stress and mitochondrial dysfunction and therefore independently drives metabolic dysfunction during DIO, regardless of dietary composition (De Mello et al., 2018). Excess energy intake, or differences in energy intake between dietary groups, therefore constitutes a confounding factor in DIO studies and may hamper the interpretation of the metabolic consequences of a given diet, or of a therapeutic or nutraceutical compound tested against the background of such a diet. However, findings from our literature survey demonstrating the poor reporting regarding dietary composition and energy intake, as well as the small number of studies reporting isocaloric intake between control and obesogenic dietary groups, provide strong evidence that these aspects are mostly not taken into consideration during either study design or data interpretation, and starkly underscores the fact that the “blunt-force-trauma” approach still predominates in animal studies of this kind. Moreover, our ranking of variables in the OPLS-DA model demonstrates that variables that are relatively easy and inexpensive to measure, such as body weight and blood glucose concentrations, may unfortunately not be very important or informative in characterising the response to obesogenic feeding. Dissection-related measurements, such as organ weight and adiposity indices, were of moderate importance in the model, while the majority of the serum-derived markers had high importance in the OPLS-DA model. Our findings should provide researchers with a guide as to which variables should be included when rodent models of DIO are characterised. Researchers should be cognisant of the diverse metabolic impact of different obesogenic diets when developing animal models of DIO, and findings should be interpreted with caution. Improved understanding of the pathophysiological mechanisms involved in DIO in animal models should contribute to the translatability of such studies within the context of human obesity.

## Data Availability

The original contributions presented in the study are included in the article/Supplementary Material, further inquiries can be directed to the corresponding author.

## Footnotes

1
https://www.envigo.com/diet-induced-obesity-custom-diets; https://researchdiets.com/opensource-diets/in-stock-diets#dio-series-diets, accessed 3 March 2022.

## Supplementary Material

Supplementary Figure S1 Receiver Operating Characteristic (ROC) curve for the OPLS-DA model. TPR, true positive rate; FPR, false positive rate. Yellow = CON; blue = OB1; red = OB2.

Supplementary Figures S2-S4 Permutation test results for the OPLS-DA model, shown per dietary group. (Figure S2) CON; (Figure S3) OB1; (Figure S4) OB2.

## References

[B1] Benevenga N. J. Calvert C. Eckhert C. D. Fahey G. C. Greger J. L. Kee C. L. (1995). Nutritional Requirements of Laboratory Animals. Subcommittee on Laboratory Animal Nutrition, Committee on Animal Nutrition, Board on Agriculture, National Research Council. Fourth Revised Edition. National Academies Press. Washington, D.C., United States.

[B2] Bylesjö M. Rantalainen M. Cloarec O. Nicholson J. K. Holmes E. Trygg J. (2006). OPLS Discriminant Analysis: Combining the Strengths of PLS-DA and SIMCA Classification. J. Chemom. 20, 341–351. 10.1002/cem.1006

[B3] Cai H. Wen Z. Meng K. Yang P. (2021). Metabolomic Signatures for Liver Tissue and Cecum Contents in High-Fat Diet-Induced Obese Mice Based on UHPLC-Q-TOF/MS. Nutr. Metab. (Lond) 18, 69. 10.1186/s12986-021-00595-8 34193189 PMC8243746

[B4] Chen R. Zeng Y. Xiao W. Zhang L. Shu Y. (2021). LC-MS-based Untargeted Metabolomics Reveals Early Biomarkers in STZ-Induced Diabetic Rats with Cognitive Impairment. Front. Endocrinol. 12, 665309. 10.3389/fendo.2021.665309 PMC8278747 34276557

[B5] De Mello A. H. Costa A. B. Engel J. D. G. Rezin G. T. (2018). Mitochondrial Dysfunction in Obesity. Life Sci. 192, 26–32. 10.1016/j.lfs.2017.11.019 29155300

[B6] Dourmashkin J. T. Chang G.-Q. Gayles E. C. Hill J. O. Fried S. K. Julien C. (2005). Different Forms of Obesity as a Function of Diet Composition. Int. J. Obes. 29, 1368–1378. 10.1038/sj.ijo.0803017 16088331

[B7] Du Toit E. F. Smith W. Muller C. Strijdom H. Stouthammer B. Woodiwiss A. J. (2008). Myocardial Susceptibility to Ischemic-Reperfusion Injury in a Prediabetic Model of Dietary-Induced Obesity. Am. J. Physiology-Heart Circulatory Physiology 294, H2336–H2343. 10.1152/ajpheart.00481.2007 18359896

[B8] Feng S. Gan L. Yang C. S. Liu A. B. Lu W. Shao P. (2018). Effects of Stigmasterol and β-Sitosterol on Nonalcoholic Fatty Liver Disease in a Mouse Model: A Lipidomic Analysis. J. Agric. Food Chem. 66, 3417–3425. 10.1021/acs.jafc.7b06146 29583004

[B9] Gooda Sahib Jambocus N. Saari N. Ismail A. Khatib A. Mahomoodally M. F. Abdul Hamid A. (2016). An Investigation into the Antiobesity Effects ofMorinda citrifoliaL. Leaf Extract in High Fat Diet Induced Obese Rats Using a1H NMR Metabolomics Approach. J. Diabetes Res. 2016, 2391592. 10.1155/2016/2391592 26798649 PMC4698747

[B10] Grima P. Guido M. Zizza A. Chiavaroli R. (2010). Sonographically Measured Perirenal Fat Thickness: an Early Predictor of Atherosclerosis in HIV-1-Infected Patients Receiving Highly Active Antiretroviral Therapy? J. Clin. Ultrasound 38, NA. 10.1002/jcu.20664 20091697

[B11] Guh D. P. Zhang W. Bansback N. Amarsi Z. Birmingham C. L. Anis A. H. (2009). The Incidence of Co-morbidities Related to Obesity and Overweight: A Systematic Review and Meta-Analysis. BMC Public Health 9, 88. 10.1186/1471-2458-9-88 19320986 PMC2667420

[B12] Kawasaki S. Aoki K. Hasegawa O. Numata K. Tanaka K. Shibata N. (2008). Sonographic Evaluation of Visceral Fat by Measuring Para- and Perirenal Fat. J. Clin. Ultrasound 36, 129–133. 10.1002/jcu.20426 18027837

[B13] Lee Y. F. Sim X. Y. Teh Y. H. Ismail M. N. Greimel P. Murugaiyah V. (2021). The Effects of High˗fat Diet and Metformin on Urinary Metabolites in Diabetes and Prediabetes Rat Models. Biotechnol. Appl. Biochem. 68, 1014–1026. 10.1002/bab.2021 32931602

[B14] Letsinger A. C. Menon R. Iyer A. R. Vellers H. L. Granados J. Z. Jayaraman A. (2020). A High Fat/high Sugar Diet Alters the Gastrointestinal Metabolome in a Sex Dependent Manner. Metabolites 10, 421. 10.3390/metabo10100421 PMC7589395 33092034

[B15] Lobstein T. Brindsen H. (2020). Obesity: Missing the 2025 Global Targets. Trends, Costs and Country Reports, March 2020. Available at: https://www.worldobesity.org/resources/resource-library/world-obesity-day-missing-the-targets-report (accessed August 19, 2020).

[B16] Lobstein T. Brindsen H. Neveux M. (2022). World Obesity Atlas 2022, March 2022. Available at: https://www.worldobesityday.org/assets/downloads/World_Obesity_Atlas_2022_WEB.pdf (accessed April 25, 2022).

[B17] Loeppke R. Taitel M. Haufle V. Parry T. Kessler R. C. Jinnett K. (2009). Health and Productivity as a Business Strategy: a Multiemployer Study. J. Occup. Environ. Med. 51, 411–428. 10.1097/JOM.0b013e3181a39180 19339899

[B18] Marques C. Meireles M. Norberto S. Leite J. Freitas J. Pestana D. (2016). High-fat Diet-Induced Obesity Rat Model: a Comparison between Wistar and Sprague-Dawley Rat. Adipocyte 5, 11–21. 10.1080/21623945.2015.1061723 27144092 PMC4836488

[B19] Matias A. Estevam W. Coelho P. Haese D. Kobi J. Lima-Leopoldo A. (2018). Differential Effects of High Sugar, High Lard or a Combination of Both on Nutritional, Hormonal and Cardiovascular Metabolic Profiles of Rodents. Nutrients 10, 1071. 10.3390/nu10081071 PMC6116051 30103515

[B20] Mediani A. Abas F. Maulidiani M. Abu Bakar Sajak A. Khatib A. Tan C. P. (2018). Metabolomic Analysis and Biochemical Changes in the Urine and Serum of Streptozotocin-Induced Normal- and Obese-Diabetic Rats. J. Physiol. Biochem. 74, 403–416. 10.1007/s13105-018-0631-3 29766441

[B21] Moreno-Fernández S. Garcés-Rimón M. Vera G. Astier J. Landrier J. Miguel M. (2018). High Fat/high Glucose Diet Induces Metabolic Syndrome in an Experimental Rat Model. Nutrients 10, 1502. 10.3390/nu10101502 PMC6213024 30322196

[B22] Muhammad N. Lembede B. W. Erlwanger K. H. (2021). Neonatal Zingerone Protects against the Development of High-Fructose Diet-Induced Metabolic Syndrome in Adult Sprague-Dawley Rats. J. Dev. Orig. Health Dis. 12, 671–679. 10.1017/S2040174420000525 32500848

[B23] Obasa Z. van Vuuren M. A. Huisamen B. Windvogel S. L. (2021). The Modulating Effects of Green Rooibos (Aspalathuslinearis) Extract on Vascular Function and Antioxidantstatus in Obese Wistar Rats. Cvja 32, 33–43. 10.5830/CVJA-2020-048 33605975 PMC9219573

[B24] Pellizzon M. A. Ricci M. R. (2018). The Common Use of Improper Control Diets in Diet-Induced Metabolic Disease Research Confounds Data Interpretation: the Fiber Factor. Nutr. Metab. (Lond) 15, 3. 10.1186/s12986-018-0243-5 29371873 PMC5769545

[B25] Peng C.-H. Lin H.-T. Chung D.-J. Huang C.-N. Wang C.-J. (2018). Mulberry Leaf Extracts Prevent Obesity-Induced NAFLD with Regulating Adipocytokines, Inflammation and Oxidative Stress. J. Food Drug Analysis 26, 778–787. 10.1016/j.jfda.2017.10.008 PMC9322217 29567249

[B26] Pezeshki A. Zapata R. C. Singh A. Yee N. J. Chelikani P. K. (2016). Low Protein Diets Produce Divergent Effects on Energy Balance. Sci. Rep. 6, 25145. 10.1038/srep25145 27122299 PMC4848496

[B27] Pickavance L. C. Tadayyon M. Widdowson P. S. Buckingham R. E. Wilding J. P. H. (1999). Therapeutic Index for Rosiglitazone in Dietary Obese Rats: Separation of Efficacy and Haemodilution. Br. J. Pharmacol. 128, 1570–1576. 10.1038/sj.bjp.0702932 10602338 PMC1571779

[B28] Pranprawit A. Wolber F. M. Heyes J. A. Molan A. L. Kruger M. C. (2013). Short-term and Long-Term Effects of Excessive Consumption of Saturated Fats And/or Sucrose on Metabolic Variables in Sprague Dawley Rats: a Pilot Study. J. Sci. Food Agric. 93, 3191–3197. 10.1002/jsfa.6240 23712415

[B29] Rana S. Blowers E. C. Natarajan A. (2015). Small Molecule Adenosine 5′-Monophosphate Activated Protein Kinase (AMPK) Modulators and Human Diseases. J. Med. Chem. 58, 2–29. 10.1021/jm401994c 25122135 PMC4864506

[B30] Sadie-Van Gijsen H. Kotzé-Hörstmann L. Huisamen B. (2020). An *In Vivo/Ex Vivo* Study Design to Investigate Effects of Chronic Conditions and Therapeutic Compounds on Adipose Stem Cells in Animal Models. Methods Mol. Biol. 2138, 101–118. 10.1007/978-1-0716-0471-7_5 32219742

[B31] Sangüesa G. Shaligram S. Akther F. Roglans N. Laguna J. C. Rahimian R. (2017). Type of Supplemented Simple Sugar, Not Merely Calorie Intake, Determines Adverse Effects on Metabolism and Aortic Function in Female Rats. Am. J. Physiology-Heart Circulatory Physiology 312, H289–H304. 10.1152/ajpheart.00339.2016 PMC5336577 27923787

[B32] Shi S. Liu Z. Xue Z. Chen X. Chu Y. (2020). A Plasma Metabonomics Study on the Therapeutic Effects of the Si-Miao-Yong-An Decoction in Hyperlipidemic Rats. J. Ethnopharmacol. 256, 112780. 10.1016/j.jep.2020.112780 32222575

[B33] Smit S. E. Manirafasha C. Marais E. Johnson R. Huisamen B. (2022). Cardioprotective Function of Green Rooibos (*Aspalathus Linearis*) Extract Supplementation in *Ex Vivo* Ischemic Prediabetic Rat Hearts. Planta Med. 88, 62–78. 10.1055/a-1239-9236 33285593

[B34] Tremmel M. Gerdtham U.-G. Nilsson P. Saha S. (2017). Economic Burden of Obesity: a Systematic Literature Review. Ijerph 14, 435. 10.3390/ijerph14040435 PMC5409636 28422077

[B35] Triba M. N. Le Moyec L. Amathieu R. Goossens C. Bouchemal N. Nahon P. (2015). PLS/OPLS Models in Metabolomics: the Impact of Permutation of Dataset Rows on the K-fold Cross-Validation Quality Parameters. Mol. Biosyst. 11, 13–19. 10.1039/c4mb00414k 25382277

[B36] Vidé J. Bonafos B. Fouret G. Benlebna M. Poupon J. Jover B. (2018). Spirulina Platensisand Silicon-Enriched Spirulina Equally Improve Glucose Tolerance and Decrease the Enzymatic Activity of Hepatic NADPH Oxidase in Obesogenic Diet-Fed Rats. Food Funct. 9, 6165–6178. 10.1039/c8fo02037j 30431036

[B37] Wallace T. M. Levy J. C. Matthews D. R. (2004). Use and Abuse of HOMA Modeling. Diabetes Care 27, 1487–1495. 10.2337/diacare.27.6.1487 15161807

[B38] Wang Y.-Q. Li S.-J. Man Y.-H. Zhuang G. (2020). Serum Metabonomics Coupled with HPLC-LTQ/orbitrap MS and Multivariate Data Analysis on the Ameliorative Effects of *Bidens Bipinnata* L. In Hyperlipidemic Rats. J. Ethnopharmacol. 262, 113196. 10.1016/j.jep.2020.113196 32730873

[B39] Whitlock G. Lewington S. Sherliker P. Clarke R. Emberson J. Halsey J. (Prospective Studies Collaboration) (2009). Body-mass Index and Cause-specific Mortality in 900 000 Adults: Collaborative Analyses of 57 Prospective Studies. Lancet 373, 1083–1096. 10.1016/S0140-6736(09)60318-4 19299006 PMC2662372

[B40] World Obesity Federation (2022). Prevalence of Obesity. Available at: https://www.worldobesity.org/about/about-obesity/prevalence-of-obesity (accessed March 10, 2022).

[B41] Worley B. Powers R. (2016). PCA as a Practical Indicator of OPLS-DA Model Reliability. Cmb 4, 97–103. 10.2174/2213235X04666160613122429 PMC4990351 27547730

[B42] Xi B. Gu H. Baniasadi H. Raftery D. (2014). Statistical Analysis and Modeling of Mass Spectrometry-Based Metabolomics Data. Methods Mol. Biol. 1198, 333–353. 10.1007/978-1-4939-1258-2_22 25270940 PMC4319703

[B43] Zhao L. Zhang Q. Ma W. Tian F. Shen H. Zhou M. (2017). A Combination of Quercetin and Resveratrol Reduces Obesity in High-Fat Diet-Fed Rats by Modulation of Gut Microbiota. Food Funct. 8, 4644–4656. 10.1039/c7fo01383c 29152632

