# Supplementary material for: Characterization and Comparison of the Divergent Metabolic Consequences of High-Sugar and High-Fat Diets in Male Wistar Rats
Source: Front Physiol. 2022 Jul 4;13:904366. doi: 10.3389/fphys.2022.904366 (PMC9290519; doi:10.3389/fphys.2022.904366)
Supplement: Supplementary file 2 [file Table1.DOCX]

**Supplementary Table 1: Additional descriptive statistics for food/energy intake and metabolic characterisation (end-point analysis) for each diet group**

|  | **Diet** | | | | | | | | | | | | | | | | | | | | | | | | | | |
| --- | --- | --- | --- | --- | --- | --- | --- | --- | --- | --- | --- | --- | --- | --- | --- | --- | --- | --- | --- | --- | --- | --- | --- | --- | --- | --- | --- |
|  | **CON** | | | | | | |  | **OB1** | | | | | | |  | **OB2** | | | | | | | | | | |
| **Variable** | NM | p-norm | Mean | Sd | Median | pctile 25 | Pctile 75 |  | NM | p-norm | Mean | Sd | Median | pctile 25 | Pctile 75 |  | NM | | p-norm | | Mean | Sd | Median | | pctile 25 | | Pctile 75 |
| baseline weight (g) | 24 | 0.839 | 187.71 | 16.03 | 187.50 | 177.25 | 201.00 |  | 24 | 0.805 | 173.21 | 17.93 | 174.50 | 163.50 | 184.25 |  | 24 | 0.571 | | 180.00 | | 14.83 | 180.50 | 172.25 | | 189.50 | |
| Final weight (g) | 23 | 0.487 | 378.26 | 38.35 | 380.00 | 355.50 | 404.50 |  | 23 | 0.852 | 430.26 | 45.08 | 432.00 | 404.00 | 457.00 |  | 24 | 0.504 | | 410.83 | | 38.63 | 409.00 | 386.00 | | 437.00 | |
| non fasted BG (mmol/L) | 23 | 0.000 | 6.71 | 1.47 | 6.30 | 6.05 | 6.80 |  | 23 | 0.611 | 6.64 | 0.57 | 6.70 | 6.30 | 6.85 |  | 24 | 0.196 | | 6.95 | | 0.43 | 6.85 | 6.70 | | 7.20 | |
| fasted BG (mmol/L) | 23 | 0.088 | 5.50 | 0.62 | 5.70 | 5.10 | 5.90 |  | 24 | 0.002 | 6.01 | 0.85 | 5.80 | 5.40 | 6.40 |  | 24 | 0.229 | | 5.94 | | 0.77 | 5.80 | 5.60 | | 6.43 | |
| BG10 | 23 | 0.068 | 6.57 | 0.82 | 6.70 | 6.15 | 6.90 |  | 22 | 0.103 | 7.02 | 1.08 | 7.05 | 6.23 | 8.08 |  | 24 | 0.164 | | 6.90 | | 0.93 | 6.95 | 6.38 | | 7.65 | |
| BG20 | 23 | 0.417 | 6.43 | 0.72 | 6.40 | 5.85 | 6.90 |  | 22 | 0.297 | 6.88 | 0.88 | 6.80 | 6.25 | 7.70 |  | 24 | 0.349 | | 6.67 | | 1.04 | 6.45 | 6.10 | | 7.25 | |
| BG30 | 23 | 0.492 | 5.99 | 0.65 | 5.90 | 5.60 | 6.35 |  | 23 | 0.057 | 6.57 | 0.91 | 6.40 | 5.85 | 6.95 |  | 24 | 0.281 | | 6.55 | | 1.34 | 6.40 | 5.78 | | 7.03 | |
| BG60 | 23 | 0.071 | 5.32 | 0.75 | 5.00 | 4.70 | 5.90 |  | 23 | 0.229 | 5.77 | 0.85 | 5.60 | 5.35 | 6.20 |  | 24 | 0.072 | | 6.08 | | 1.09 | 5.90 | 5.38 | | 6.53 | |
| BG90 | 23 | 0.910 | 5.24 | 0.55 | 5.30 | 4.90 | 5.50 |  | 23 | 0.004 | 5.74 | 0.69 | 5.40 | 5.25 | 6.10 |  | 24 | 0 | | 5.66 | | 1.22 | 5.45 | 5.03 | | 5.90 | |
| BG120 | 23 | 0.029 | 5.22 | 0.60 | 5.00 | 4.80 | 5.65 |  | 23 | 0.371 | 5.85 | 0.66 | 6.00 | 5.40 | 6.30 |  | 24 | 0 | | 5.51 | | 1.23 | 5.25 | 4.70 | | 5.90 | |
| OGTT AUC (A.U.) | 23 | 0.106 | 194.18 | 54.60 | 183.70 | 151.10 | 241.90 |  | 23 | 0.169 | 256.74 | 64.42 | 259.10 | 198.20 | 294.05 |  | 24 | 0.015 | | 254.34 | | 129.25 | 232.50 | 170.40 | | 286.73 | |
| Adiponectin (ng/mL) | 23 | 0.016 | 55.91 | 18.39 | 55.38 | 43.82 | 61.72 |  | 24 | 0.215 | 88.89 | 26.29 | 90.44 | 68.04 | 111.18 |  | 23 | 0.105 | | 60.81 | | 22.42 | 58.81 | 46.88 | | 69.43 | |
| Liver weight (g) | 23 | 0.190 | 12.03 | 1.51 | 11.58 | 10.85 | 13.12 |  | 23 | 0.231 | 11.99 | 1.42 | 11.69 | 10.83 | 13.12 |  | 24 | 0.094 | | 15.50 | | 1.70 | 15.79 | 14.87 | | 16.49 | |
| PV weight (g) | 23 | 0.209 | 0.89 | 0.30 | 0.87 | 0.69 | 1.05 |  | 23 | 0.188 | 1.56 | 0.50 | 1.66 | 1.15 | 1.80 |  | 24 | 0.268 | | 1.46 | | 0.52 | 1.35 | 1.06 | | 1.82 | |
| VAI (%) | 23 | 0.099 | 0.47 | 0.14 | 0.49 | 0.41 | 0.51 |  | 23 | 0.354 | 0.72 | 0.20 | 0.74 | 0.57 | 0.85 |  | 24 | 0.108 | | 0.70 | | 0.21 | 0.66 | 0.57 | | 0.82 | |
| Pancreas weight (g) | 23 | 0.001 | 0.63 | 0.16 | 0.56 | 0.53 | 0.71 |  | 20 | 0.641 | 0.62 | 0.15 | 0.61 | 0.55 | 0.68 |  | 22 | 0.817 | | 0.43 | | 0.08 | 0.44 | 0.37 | | 0.48 | |
| TG (mmol/L) | 18 | 0.000 | 0.67 | 0.26 | 0.61 | 0.52 | 0.73 |  | 21 | 0.688 | 1.24 | 0.35 | 1.28 | 1.00 | 1.39 |  | 23 | 0.235 | | 1.27 | | 0.42 | 1.17 | 0.93 | | 1.55 | |
| TC (mmol/L) | 18 | 0.083 | 1.84 | 0.28 | 1.80 | 1.63 | 1.98 |  | 21 | 0.019 | 1.67 | 0.25 | 1.60 | 1.50 | 1.80 |  | 23 | 0.655 | | 2.95 | | 0.65 | 3.00 | 2.40 | | 3.30 | |
| HDL-C (mmol/L) | 18 | 0.114 | 1.18 | 0.22 | 1.10 | 1.00 | 1.30 |  | 21 | 0.093 | 1.01 | 0.13 | 1.00 | 0.90 | 1.10 |  | 23 | 0.541 | | 1.13 | | 0.18 | 1.10 | 1.00 | | 1.25 | |
| LDL-C (mmol/L) | 18 | 0.000 | 0.11 | 0.06 | 0.10 | 0.10 | 0.10 |  | 21 | 0 | 0.13 | 0.15 | 0.10 | 0.10 | 0.10 |  | 23 | 0.967 | | 1.17 | | 0.49 | 1.10 | 0.85 | | 1.50 | |
| ALT (IU/L) | 18 | 0.009 | 38.22 | 8.80 | 36.00 | 32.50 | 41.50 |  | 21 | 0 | 42.38 | 14.24 | 39.00 | 36.00 | 40.00 |  | 23 | 0 | | 145.09 | | 94.80 | 116.00 | 92.00 | | 150.00 | |
| AST (IU/L) | 18 | 0.065 | 104.44 | 18.41 | 96.00 | 91.25 | 120.00 |  | 21 | 0 | 95.29 | 18.71 | 90.00 | 84.00 | 100.00 |  | 23 | 0 | | 148.44 | | 57.56 | 139.00 | 115.00 | | 159.50 | |
| Insulin (ng/mL) | 23 | 0.061 | 1.96 | 1.16 | 1.64 | 1.23 | 2.73 |  | 24 | 0.091 | 4.11 | 1.93 | 3.65 | 2.79 | 4.97 |  | 22 | 0.095 | | 3.15 | | 1.88 | 2.71 | 1.88 | | 4.06 | |
| HOMA2 IR (A.U.) | 23 | 0.232 | 6.62 | 3.53 | 5.99 | 4.49 | 8.94 |  | 24 | 0.539 | 11.18 | 3.52 | 10.61 | 8.75 | 14.24 |  | 22 | 0.923 | | 9.08 | | 4.00 | 8.82 | 6.61 | | 12.27 | |
| HOMA2 %B (A.U.) | 23 | 0.058 | 299.10 | 110.61 | 259.50 | 215.45 | 381.40 |  | 24 | 0.6 | 418.13 | 120.39 | 413.90 | 329.45 | 528.53 |  | 22 | 0.091 | | 343.52 | | 145.05 | 322.95 | 252.70 | | 395.03 | |
| HOMA2 %S (A.U.) | 23 | 0.000 | 21.24 | 16.45 | 16.70 | 11.20 | 22.30 |  | 24 | 0.001 | 10.14 | 4.40 | 9.45 | 7.03 | 11.43 |  | 22 | 0 | | 14.62 | | 11.06 | 11.35 | 8.15 | | 15.13 | |
| TNF-α (pg/mL) | 23 | 0.200 | 0.70 | 0.52 | 0.58 | 0.21 | 1.17 |  | 23 | 0.004 | 0.51 | 0.51 | 0.39 | 0.00 | 0.97 |  | 24 | 0 | | 0.51 | | 0.93 | 0.00 | 0.00 | | 0.74 | |
| Leptin (pg/mL) | 23 | 0.012 | 16344.25 | 5648.73 | 14674.90 | 13175.35 | 18181.05 |  | 23 | 0.023 | 27318.64 | 9312.39 | 25510.20 | 22123.30 | 28083.25 |  | 24 | 0.01 | | 14089.67 | | 7489.74 | 13258.25 | 8244.87 | | 17030.35 | |
| IL-18 (pg/mL) | 23 | 0.003 | 173.18 | 77.36 | 143.93 | 125.28 | 198.63 |  | 23 | 0.529 | 186.23 | 54.78 | 176.91 | 150.92 | 211.71 |  | 24 | 0.021 | | 292.64 | | 139.80 | 286.08 | 204.49 | | 332.68 | |

Abbreviations: ALT: alanine transaminase; AST: aspartate transaminase; BG: blood glucose; HDL-C: high-density lipoprotein cholesterol; HOMA: homeostatic model assessment; IL-18: interleukin-18; IR: insulin resistance; LDL-C: low-density lipoprotein cholesterol; OGTT AUC: oral glucose tolerance test area under the curve; PV: perirenal visceral fat; TC: total cholesterol; TG: total triacylglycerol; TNF-α: tumour necrosis factor-alpha; VAI: visceral adiposity index. BG10-BG120 refer to blood glucose levels at various time-points (in minutes) during the OGTT, and are reported as mmol/L. Where n < 24, this resulted from samples not being available for analysis. No data points were excluded as outliers.
