# Supplementary figures and images for: Characterization and Comparison of the Divergent Metabolic Consequences of High-Sugar and High-Fat Diets in Male Wistar Rats
Source: Front Physiol. 2022 Jul 4;13:904366. doi: 10.3389/fphys.2022.904366 (PMC9290519; doi:10.3389/fphys.2022.904366)

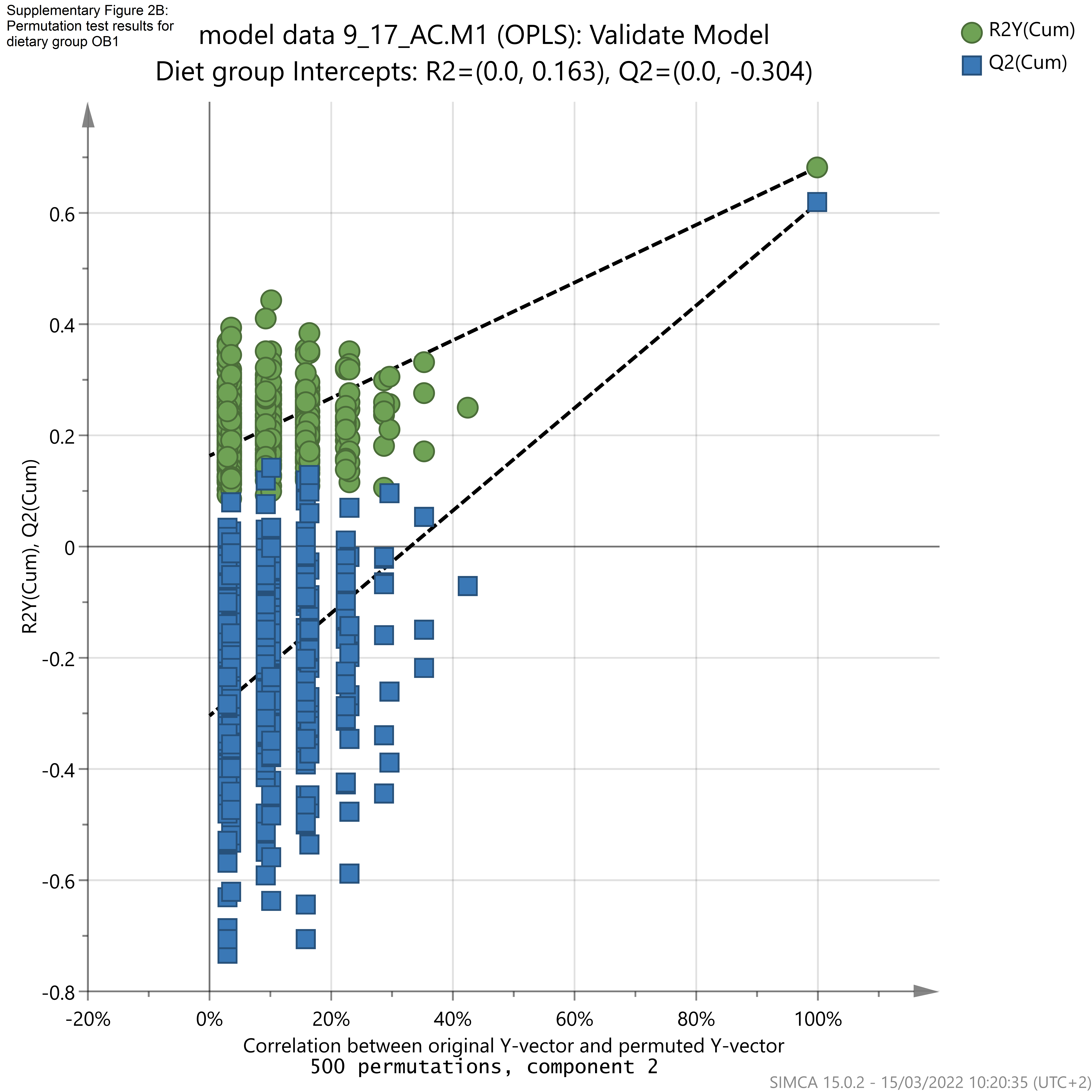

Supplement: Supplementary file 3 [file Image3.TIF]

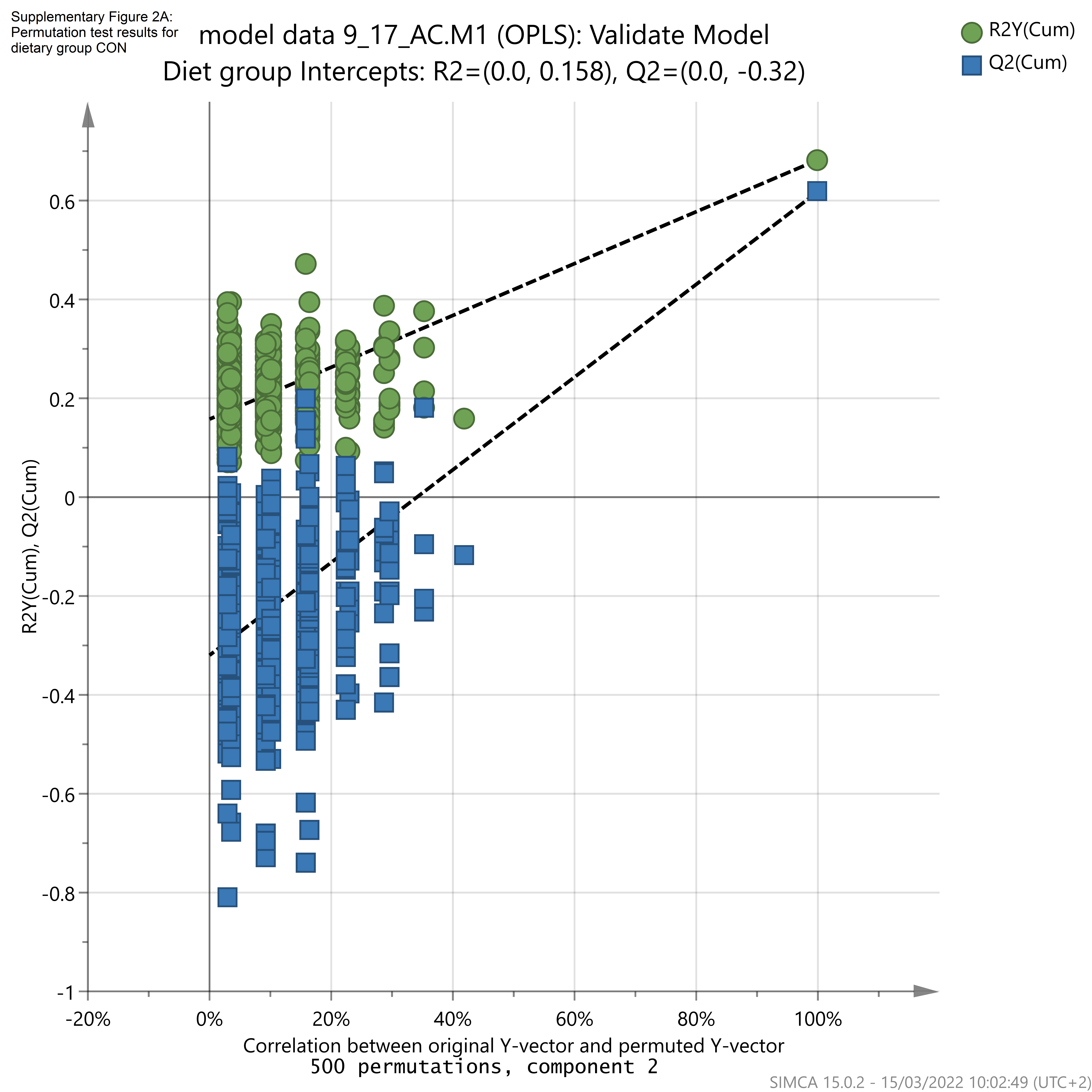

Supplement: Supplementary file 4 [file Image2.TIF]

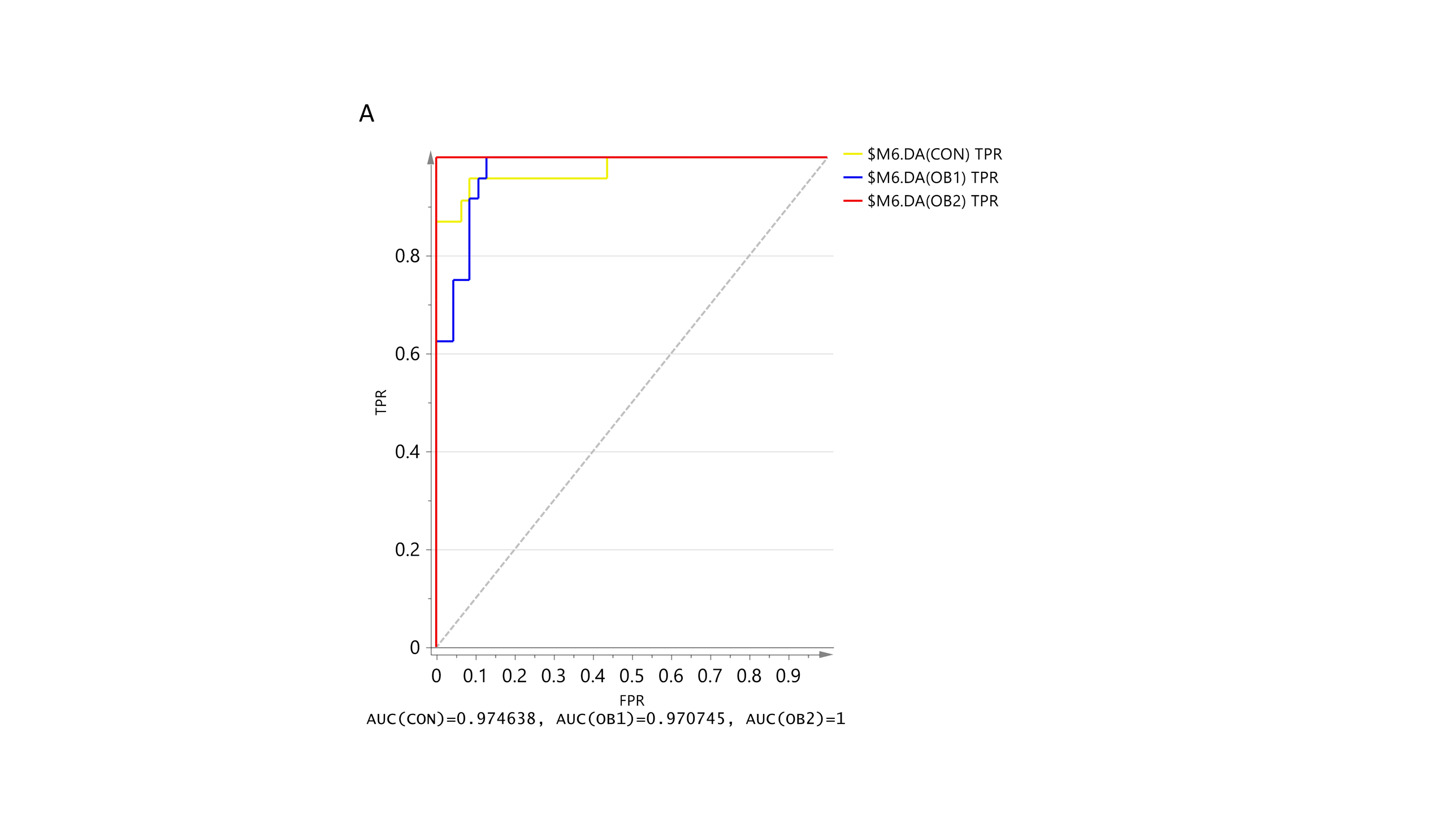

Supplement: Supplementary file 5 [file Image1.TIF]

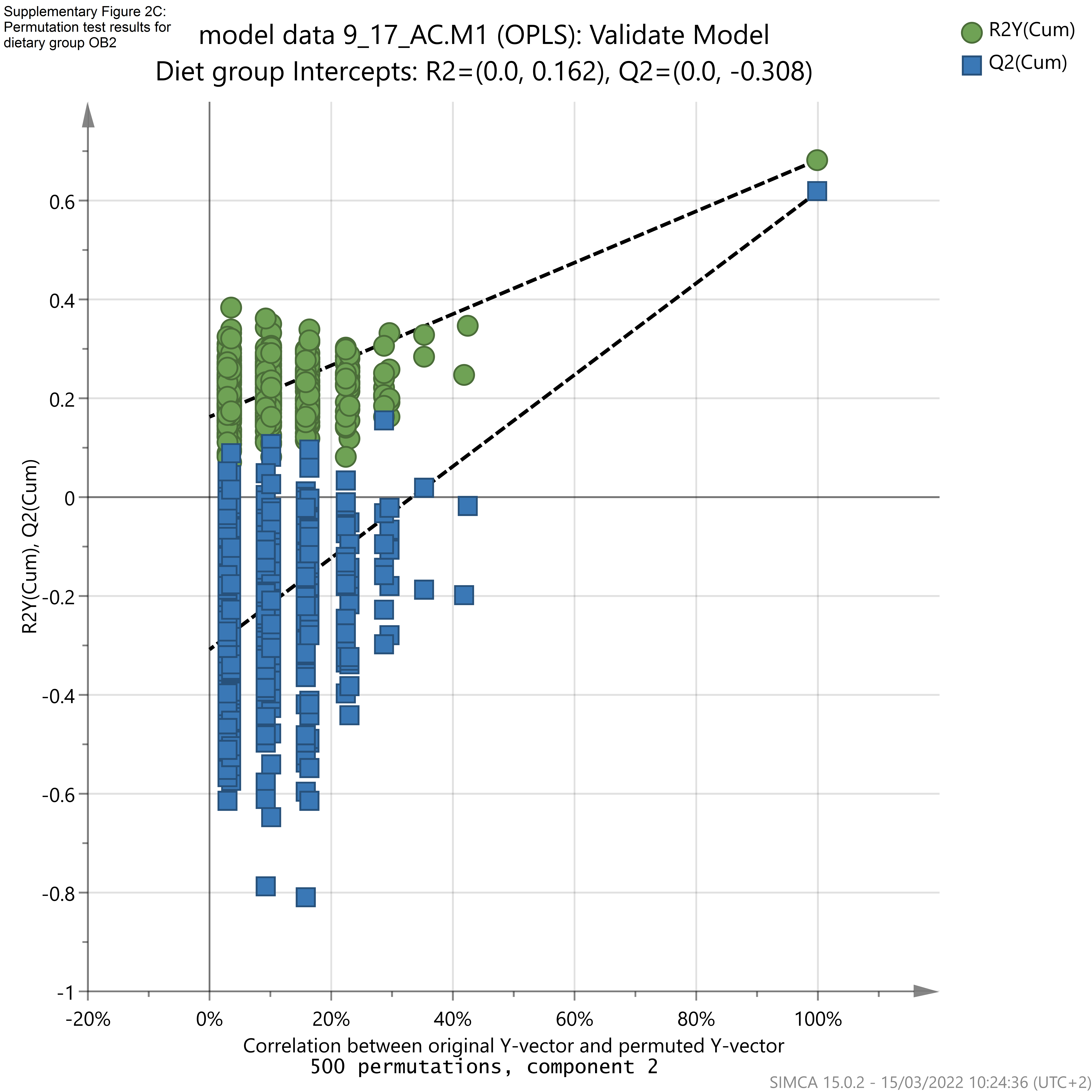

Supplement: Supplementary file 8 [file Image4.TIFF]
