# Supplementary material for: Characterization and Comparison of the Divergent Metabolic Consequences of High-Sugar and High-Fat Diets in Male Wistar Rats
Source: Front Physiol. 2022 Jul 4;13:904366. doi: 10.3389/fphys.2022.904366 (PMC9290519; doi:10.3389/fphys.2022.904366)
Supplement: Supplementary file 7 [file Table3.DOCX]

**Supplementary Table 3: OPLS-DA model parameters**

| Component | Model | Predictive | P1 | P2 | Orthogonal  in X(OPLS) | O1 |
| --- | --- | --- | --- | --- | --- | --- |
| R2X |  |  | 0.161 | 0.159 |  | 0.172 |
| R2X(cum) | 0.492 | 0.319 | 0.161 | 0.319 | 0.172 | 0.172 |
| Eigenvalue |  |  | 4.66 | 4.6 |  | 5.01 |
| R2 |  |  | 0.409 | 0.304 |  | 0 |
| R2(cum) | 0.713 | 0.713 | 0.409 | 0.713 | 0 | 0 |
| Q2 |  |  | 0.37 | 0.293 |  |  |
| Limit |  |  | 0.01 | 0.01 |  |  |
| Q2(cum) | 0.663 | 0.663 | 0.37 | 0.663 |  |  |
| R2Y |  |  | 0.503 | 0.497 |  |  |
| R2Y(cum) | 1 | 1 | 0.503 | 1 |  |  |
| Eigenvalue Y |  |  | 1.51 | 1.49 |  |  |
| Significance |  |  | R1 | R1 |  | R1 |
