# Supplementary material for: Characterization and Comparison of the Divergent Metabolic Consequences of High-Sugar and High-Fat Diets in Male Wistar Rats
Source: Front Physiol. 2022 Jul 4;13:904366. doi: 10.3389/fphys.2022.904366 (PMC9290519; doi:10.3389/fphys.2022.904366)
Supplement: Supplementary file 9 [file DataSheet1.docx]

Abdel-Sattar, E., Mehanna, E.T., El-Ghaiesh, S.H., Mohammad, H.M.F., Elgendy, H.A., Zaitone, S.A. (2018). Pharmacological action of a pregnane glycoside, Russelioside B, in dietary obese rats: Impact on weight gain and energy expenditure. Front. Pharmacol. 9, 990. doi: 10.3389/fphar.2018.00990.

Aborehab, N.M., El Bishbishy, M.H., Waly, N.E. (2016). Resistin mediates tomato and broccoli extract effects on glucose homeostasis in high fat diet-induced obesity in rats. BMC Complement. Altern. Med. 16, 225. doi: 10.1186/s12906-016-1203-0.

Ajiboye, T.O., Hussaini, A.A., Nafiu, B.Y., Ibitoye, O.B. (2017). Aqueous seed extract of *Hunteria umbellata* (K. Schum.) Hallier f. (Apocynaceae) palliates hyperglycemia, insulin resistance, dyslipidemia, inflammation and oxidative stress in high-fructose diet-induced metabolic syndrome in rats. J. Ethnopharmacol. 198, 184-193. doi: 10.1016/j.jep.2016.11.043.

Al-Sawalha, N.A., Alshogran, O.Y., Awawdeh, M.S., Almomani, B.A. (2019). The effects of l-Carnosine on development of metabolic syndrome in rats. Life Sci. 237, 116905. doi: 10.1016/j.lfs.2019.116905.

Amor, S., González-Hedström, D., Martín-Carro, B., Inarejos-García, A.M., Almodóvar, P., Prodanov, M., García-Villalón, A.L., Granado, M. (2019). Beneficial effects of an aged black garlic extract in the metabolic and vascular alterations induced by a high fat/sucrose diet in male rats. Nutrients 11, 153. doi: 10.3390/nu11010153.

Aslam, M., Madhu, S.V. (2017). Development of metabolic syndrome in high-sucrose diet fed rats is not associated with decrease in adiponectin levels. Endocrine 58, 59-65. doi: 10.1007/s12020-017-1403-5

Barragán-Zarate, G.S., Alexander-Aguilera, A., Lagunez-Rivera, L., Solano, R., Soto-Rodríguez, I. (2021). Bioactive compounds from *Prosthechea karwinskii* decrease obesity, insulin resistance, pro-inflammatory status, and cardiovascular risk in Wistar rats with metabolic syndrome. J. Ethnopharmacol. 279, 114376. doi: 10.1016/j.jep.2021.114376

Boldarine, V.T., Joyce, E., Pedroso, A.P., Telles, M.M., Oyama, L.M., Bueno, A.A., Ribeiro, E.B. (2021). Oestrogen replacement fails to fully revert ovariectomy-induced changes in adipose tissue monoglycerides, diglycerides and cholesteryl esters of rats fed a lard-enriched diet. Sci. Rep. 11, 3841. doi: 10.1038/s41598-021-82837-6.

Bunbupha, S., Apaijit, K., Maneesai, P., Prasarttong, P., Pakdeechote, P. (2020). Nobiletin ameliorates high-fat diet-induced vascular and renal changes by reducing inflammation with modulating AdipoR1 and TGF-β1 expression in rats. Life Sci. 260, 118398. doi: 10.1016/j.lfs.2020.118398.

Bunbupha, S., Prasarttong, P., Poasakate, A., Maneesai, P., Pakdeechote, P. (2021). Imperatorin alleviates metabolic and vascular alterations in high-fat/high-fructose diet-fed rats by modulating adiponectin receptor 1, eNOS, and p47phox expression. Eur. J. Pharmacol. 899, 174010. doi: 10.1016/j.ejphar.2021.174010.

Chacińska, M., Zabielski, P., Książek, M., Szałaj, P., Jarząbek, K., Kojta, I., Chabowski, A., Błachnio-Zabielska, A.U. (2019). The impact of OMEGA-3 fatty acids supplementation on insulin resistance and content of adipocytokines and biologically active lipids in adipose tissue of high-fat diet fed rats. Nutrients 11, 835. doi: 10.3390/nu11040835.

Cheng, H.S., Yaw, H.P., Ton, S.H., Choy, S.M., Kong, J.M., Abdul Kadir, K. (2016). Glycyrrhizic acid prevents high calorie diet-induced metabolic aberrations despite the suppression of peroxisome proliferator-activated receptor γ expression. Nutrition 32, 995-1001. doi: 10.1016/j.nut.2016.02.002.

Das, S., Choudhuri, D. (2020). Dietary calcium regulates the insulin sensitivity by altering the adipokine secretion in high fat diet induced obese rats. Life Sci 250, 117560. doi: 10.1016/j.lfs.2020.117560

de Las Heras, N., Valero-Muñoz, M., Martín-Fernández, B., Ballesteros, S., López-Farré, A., Ruiz-Roso, B., Lahera, V. (2017). Molecular factors involved in the hypolipidemic- and insulin-sensitizing effects of a ginger (*Zingiber officinale* Roscoe) extract in rats fed a high-fat diet. Appl Physiol Nutr Metab 42, 209-215. doi: 10.1139/apnm-2016-0374.

El-Shazly, S.A., Ahmed, M.M., Al-Harbi, M.S., Alkafafy, M.E., El-Sawy, H.B., Amer, S.A.M. (2018). Physiological and molecular study on the anti-obesity effects of pineapple (*Ananas comosus*) juice in male Wistar rat. Food Sci. Biotechnol. 27, 1429-1438. doi: 10.1007/s10068-018-0378-1.

El-Zeftawy, M., Ali, S.A.M., Salah, S., Hafez, H.S. (2020). The functional nutritional and regulatory activities of calcium supplementation from eggshell for obesity disorders management. J. Food Biochem. 44, e13313. doi: 10.1111/jfbc.13313.

Fakhoury-Sayegh, N., Trak-Smayra, V., Sayegh, R., Haidar, F., Obeid, O., Asmar, S., Khazzaka, A. (2019). Fructose threshold for inducing organ damage in a rat model of nonalcoholic fatty liver disease. Nutr. Res. 62, 101-112. doi: 10.1016/j.nutres.2018.11.003.

Fallah, H., Akbari, H., Abolhassani, M., Mohammadi, A., Gholamhosseinian, A. (2017). *Berberis integerrima* ameliorates insulin resistance in high-fructose-fed insulin-resistant rats. Iran. J. Basic Med. Sci. 20, 1093-1101. doi: 10.22038/IJBMS.2017.9409.

Fiorino, P., Américo, A.L.V., Muller, C.R., Evangelista, F.S., Santos, F., Leite, A.P.O., Farah V. (2016). Exposure to high-fat diet since post-weaning induces cardiometabolic damage in adult rats. Life Sci. 160, 12-17. doi: 10.1016/j.lfs.2016.07.001.

Gomaa, A.A., Farghaly, H.S.M., El-Sers, D.A., Farrag, M.M., Al-Zokeim, N.I. (2019). Inhibition of adiposity and related metabolic disturbances by polyphenol-rich extract of *Boswellia serrata* gum through alteration of adipo/cytokine profiles. Inflammopharmacology 27, 549-559. doi: 10.1007/s10787-018-0519-4.

Grasa-López, A., Miliar-García, Á., Quevedo-Corona, L., Paniagua-Castro, N., Escalona-Cardoso, G., Reyes-Maldonado, E., Jaramillo-Flores, M.E. (2016). *Undaria pinnatifida* and fucoxanthin ameliorate lipogenesis and markers of both inflammation and cardiovascular dysfunction in an animal model of diet-induced obesity. Mar. Drugs 14, 148. doi: 10.3390/md14080148.

Grycel, S., Markowski, A.R., Hady, H.R., Zabielski, P., Kojta, I., Imierska, M., Górski, J., Blachnio-Zabielska, A.U. (2019). Metformin treatment affects adipocytokine secretion and lipid composition in adipose tissues of diet-induced insulin-resistant rats. Nutrition 63-64, 126-133. doi: 10.1016/j.nut.2019.01.019.

Gumede, N.M., Lembede, B.W., Brooksbank, R.L., Erlwanger, K.H., Chivandi, E. (2020). β-Sitosterol shows potential to protect against the development of high-fructose diet-induced metabolic dysfunction in female rats. J. Med. Food 23, 367-374. doi: 10.1089/jmf.2019.0120.

Han, Y., Lu, Z., Chen, S., Zhong, C., Yan, M., Wang, H., Meng, M., Liu, M. (2021). Abdominal massage alleviates skeletal muscle insulin resistance by regulating the AMPK/SIRT1/PGC-1α signaling pathway. Cell. Biochem. Biophys. 79, 895-903. doi: 10.1007/s12013-021-00983-0.

Heo, M.G., Choung, S.Y. (2018). Anti-obesity effects of *Spirulina maxima* in high fat diet induced obese rats via the activation of AMPK pathway and SIRT1. Food Funct. 9, 4906-4915. doi: 10.1039/c8fo00986d.

Ibitoye, O.B., Ajiboye, T.O. (2018). Dietary phenolic acids reverse insulin resistance, hyperglycaemia, dyslipidaemia, inflammation and oxidative stress in high-fructose diet-induced metabolic syndrome rats. Arch. Physiol. Biochem. 124, 410-417. doi: 10.1080/13813455.2017.1415938.

Jambocus, N.G.S., Ismail, A., Khatib, A., Mahomoodally, F., Saari, N., Mumtaz, M.W., Hamid, A.A. (2017). *Morinda citrifolia* L. leaf extract prevent weight gain in Sprague-Dawley rats fed a high fat diet. Food Nutr. Res. 61, 1338919. doi: 10.1080/16546628.2017.1338919.

Jang, H.H., Nam, S.Y., Kim, M.J., Kim, J.B., Choi, J.S., Kim, H.R., Lee, Y.M. (2017). *Agrimonia pilosa* Ledeb. aqueous extract improves impaired glucose tolerance in high-fat diet-fed rats by decreasing the inflammatory response. BMC Complement. Altern. Med. 17, 442. doi: 10.1186/s12906-017-1949-z.

Khaleel, E.F., Abdel-Aleem, G.A. (2019). Obestatin protects and reverses nonalcoholic fatty liver disease and its associated insulin resistance in rats via inhibition of food intake, enhancing hepatic adiponectin signaling, and blocking ghrelin acylation. Arch. Physiol. Biochem. 125, 64-78. doi: 10.1080/13813455.2018.1437638.

Kim, J., Kim, C.S., Jo, K., Lee, I.S., Kim, J.H., Kim, J.S. (2020). POCU1b, the n-butanol soluble fraction of Polygoni Cuspidati Rhizoma et Radix, attenuates obesity, non-alcoholic fatty liver, and insulin resistance via inhibitions of pancreatic lipase, cAMP-dependent PDE activity, AMPK activation, and SOCS-3 suppression. Nutrients 12, 3612. doi: 10.3390/nu12123612.

Lavet, C., Martin, A., Linossier, M.T., Vanden Bossche, A., Laroche, N., Thomas, M., Gerbaix, M., Ammann, P., Fraissenon, A., Lafage-Proust, M.H., Courteix, D., Vico, L. (2016). Fat and sucrose intake induces obesity-related bone metabolism disturbances: Kinetic and reversibility studies in growing and adult rats. J. Bone Miner. Res. 31, 98-115. doi: 10.1002/jbmr.2596.

Lenquiste, S.A., de Almeida Lamas, C., da Silva Marineli, R., Moraes, É.A., Borck, P.C., Camargo, R.L., Quitete, V.H.A.C., Carneiro, E.M., Junior, M.R.M. (2019). Jaboticaba peel powder and jaboticaba peel aqueous extract reduces obesity, insulin resistance and hepatic fat accumulation in rats. Food Res. Int. 120, 880-887. doi: 10.1016/j.foodres.2018.11.053.

Lima, G.C., Vuolo, M.M., Batista, Â.G., Dragano, N.R., Solon, C., Maróstica Junior, M.R. (2016). *Passiflora edulis* peel intake improves insulin sensitivity, increasing incretins and hypothalamic satietogenic neuropeptide in rats on a high-fat diet. Nutrition 32, 863-70. doi: 10.1016/j.nut.2016.01.014.

Mahmoudi, M., Charradi, K., Limam, F., Aouani, E. (2018). Grape seed and skin extract as an adjunct to xenical therapy reduces obesity, brain lipotoxicity and oxidative stress in high fat diet fed rats. Obes. Res. Clin. Pract. 12(Suppl 2), 115-126. doi: 10.1016/j.orcp.2016.04.006.

Marques, C., Meireles, M., Norberto, S., Leite, J., Freitas, J., Pestana, D., Faria, A., Calhau, C. (2016). High-fat diet-induced obesity Rat model: a comparison between Wistar and Sprague-Dawley Rat. Adipocyte 5, 11-21. doi: 10.1080/21623945.2015.1061723.

Mazzoli, A., Spagnuolo, M.S., Gatto, C., Nazzaro, M., Cancelliere, R., Crescenzo, R., Iossa, S., Cigliano, L. (2020). Adipose tissue and brain metabolic responses to Western diet-Is there a similarity between the two? Int. J. Mol. Sci. 21, 786. doi: 10.3390/ijms21030786.

Mehanna, E.T., Barakat, B.M., ElSayed, M.H., Tawfik, M.K. (2018). An optimized dose of raspberry ketones controls hyperlipidemia and insulin resistance in male obese rats: Effect on adipose tissue expression of adipocytokines and Aquaporin 7. Eur. J. Pharmacol. 832, 81-89. doi: 10.1016/j.ejphar.2018.05.028.

Mehrdoost, S., Yaghmaei, P., Jafary, H., Ebrahim-Habibi, A. (2021). The therapeutic effects of berberine plus sitagliptin in a rat model of fatty liver disease. Iran. J. Basic Med. Sci. 24, 451-459. doi: 10.22038/ijbms.2021.52239.11822.

Meriga, B., Naidu, P.B., Muniswamy, G., Kumar, G.H., Naik, R.R., Pothani, S. (2017). Ethanolic fraction of *Terminalia tomentosa* attenuates biochemical and physiological derangements in diet induced obese rat model by regulating key lipid metabolizing enzymes and adipokines. Pharmacogn. Mag. 13, 385-392. doi: 10.4103/0973-1296.208871.

Meriga, B., Parim, B., Chunduri, V.R., Naik, R.R., Nemani, H., Suresh, P., Ganapathy, S., Sathibabu Uddandrao, V.V. (2017). Antiobesity potential of Piperonal: promising modulation of body composition, lipid profiles and obesogenic marker expression in HFD-induced obese rats. Nutr. Metab. (Lond.) 14, 72. doi: 10.1186/s12986-017-0228-9.

Metwally, F.M., Rashad, H., Mahmoud, A.A. (2019). *Morus alba* L. diminishes visceral adiposity, insulin resistance, behavioral alterations via regulation of gene expression of leptin, resistin and adiponectin in rats fed a high-cholesterol diet. Physiol. Behav. 201, 1-11. doi: 10.1016/j.physbeh.2018.12.010.

Mohaqiq, Z., Moossavi, M., Hemmati, M., Kazemi, T., Mehrpour, O. (2020). Antioxidant properties of Saffron Stigma and Petals: a potential therapeutic approach for insulin resistance through an insulin-sensitizing adipocytokine in high-calorie diet rats. Int. J. Prev. Med. 11, 184. doi: 10.4103/ijpvm.IJPVM_275_19.

Moreno-Fernández, S., Garcés-Rimón, M., Vera, G., Astier, J., Landrier, J.F., Miguel, M. (2018). High fat/high glucose diet induces Metabolic Syndrome in an experimental rat model. Nutrients 10, 1502. doi: 10.3390/nu10101502.

Muhammad, N., Lembede, B.W., Erlwanger, K.H. (2021). Neonatal zingerone protects against the development of high-fructose diet-induced metabolic syndrome in adult Sprague-Dawley rats. J. Dev. Orig. Health Dis. 12, 671-679. doi: 10.1017/S2040174420000525.

Nour, O.A., Ghoniem, H.A., Nader, M.A., Suddek, G.M. (2021). Impact of protocatechuic acid on high fat diet-induced metabolic syndrome sequelae in rats. Eur. J. Pharmacol. 907, 174257. doi: 10.1016/j.ejphar.2021.174257.

Ohashi, K., Ohta, Y., Ishikawa, H., Kitagawa, A. (2021). Orally administered octacosanol improves some features of high fructose-induced metabolic syndrome in rats. J. Clin. Biochem. Nutr. 68, 58-66. doi: 10.3164/jcbn.20-48.

Pascual-Serrano, A., Arola-Arnal, A., Suárez-García, S., Bravo, F.I., Suárez, M., Arola, L., Bladé, C. (2017). Grape seed proanthocyanidin supplementation reduces adipocyte size and increases adipocyte number in obese rats. Int. J. Obes. (Lond.) 41, 1246-1255. doi: 10.1038/ijo.2017.90.

Prasatthong, P., Meephat, S., Rattanakanokchai, S., Khamseekaew, J., Bunbupha, S., Prachaney, P., Maneesai, P., Pakdeechote, P. (2021). Galangin resolves cardiometabolic disorders through modulation of AdipoR1, COX-2, and NF-κB expression in rats fed a high-fat diet. Antioxidants. (Basel) 10, 769. doi: 10.3390/antiox10050769.

Pruszyńska-Oszmałek, E., Wojciechowska, M., Sassek, M., Krauss, H., Leciejewska, N., Szczepankiewicz, D., Ślósarz, P., Nogowski, L., Kołodziejski, P.A. (2021). The long-term effects of high-fat and high-protein diets on the metabolic and endocrine activity of adipocytes in rats. Biology. (Basel) 10:339. doi: 10.3390/biology10040339.

Putakala, M., Gujjala, S., Nukala, S., Desireddy, S. (2017). Beneficial Effects of *Phyllanthus amarus* against high fructose diet induced insulin resistance and hepatic oxidative stress in male Wistar rats. Appl. Biochem. Biotechnol. 183, 744-764. doi: 10.1007/s12010-017-2461-0.

Rahman, H.A., Sahib, N.G., Saari, N., Abas, F., Ismail, A., Mumtaz, M.W., Hamid, A.A. (2017). Anti-obesity effect of ethanolic extract from *Cosmos caudatus* Kunth leaf in lean rats fed a high fat diet. BMC Complement. Altern. Med. 17, 122. doi: 10.1186/s12906-017-1640-4.

Rameshreddy, P., Uddandrao, V.V.S., Brahmanaidu, P., Vadivukkarasi, S., Ravindarnaik, R., Suresh, P., Swapna, K., Kalaivani, A., Parvathi, P., Tamilmani, P., Saravanan, G. (2018). Obesity-alleviating potential of asiatic acid and its effects on ACC1, UCP2, and CPT1 mRNA expression in high fat diet-induced obese Sprague-Dawley rats. Mol. Cell. Biochem. 442, 143-154. doi: 10.1007/s11010-017-3199-2.

Ramos-Romero, S., Molinar-Toribio, E., Pérez-Jiménez, J., Taltavull, N., Dasilva, G., Romeu, M., Medina, I., Torres, J.L. (2016). The combined action of omega-3 polyunsaturated fatty acids and grape proanthocyanidins on a rat model of diet-induced metabolic alterations. Food Funct. 7, 3516-3523. doi: 10.1039/c6fo00679e

Rocha-Rodrigues, S., Gonçalves, I.O., Beleza, J., Ascensão, A., Magalhães, J. (2018). Physical exercise mitigates high-fat diet-induced adiposopathy and related endocrine alterations in an animal model of obesity. J. Physiol. Biochem. 74, 235-246. doi: 10.1007/s13105-018-0609-1.

Rubio-Ruiz, M.E., Guarner-Lans, V., Cano-Martínez, A., Díaz-Díaz, E., Manzano-Pech, L., Gamas-Magaña, A., Castrejón-Tellez, V., Tapia-Cortina, C., Pérez-Torres, I. (2019). Resveratrol and quercetin administration improves antioxidant DEFENSES and reduces fatty liver in metabolic syndrome rats. Molecules. 24, 1297. doi: 10.3390/molecules24071297.

Sangüesa, G., Shaligram, S., Akther, F., Roglans, N., Laguna, J.C., Rahimian, R., Alegret, M. (2017). Type of supplemented simple sugar, not merely calorie intake, determines adverse effects on metabolism and aortic function in female rats. Am. J. Physiol. Heart Circ. Physiol. 312, H289-H304. doi: 10.1152/ajpheart.00339.2016.

Sarega, N., Imam, M.U., Esa, N.M., Zawawi, N., Ismail, M. (2016). Effects of phenolic-rich extracts of *Clinacanthus nutan*s on high fat and high cholesterol diet-induced insulin resistance. BMC Complement. Altern. Med. 16, 88. doi: 10.1186/s12906-016-1049-5.

Shao, S., Zhang, X., Zhang, M. (2016). Inhibition of 11β-hydroxysteroid dehydrogenase type 1 ameliorates obesity-related insulin resistance. Biochem. Biophys. Res. Commun. 478, 474-480. doi: 10.1016/j.bbrc.2016.06.015.

Tan, L., Song, A., Ren, L., Wang, C., Song, G. (2020). Effect of pioglitazone on skeletal muscle lipid deposition in the insulin resistance rat model induced by high fructose diet under AMPK signaling pathway. Saudi J. Biol. Sci. 27, 1317-1323. doi: 10.1016/j.sjbs.2020.03.014.

Tanajak, P., Pintana, H., Siri-Angkul, N., Khamseekaew, J., Apaijai, N., Chattipakorn, S.C., Chattipakorn, N. (2017). Vildagliptin and caloric restriction for cardioprotection in pre-diabetic rats. J. Endocrinol. 232, 189-204. doi: 10.1530/JOE-16-0406.

Tinkov, A.A., Gatiatulina, E.R., Popova, E.V., Polyakova, V.S., Skalnaya, A.A., Agletdinov, E.F., Nikonorov, A.A., Skalny, A.V. (2017). Early high-fat feeding induces alteration of trace element content in tissues of juvenile male Wistar rats. Biol. Trace Elem. Res. 175, 367-374. doi: 10.1007/s12011-016-0777-1.

Tinkov, A.A., Popova, E.V., Gatiatulina, E.R., Skalnaya, A.A., Yakovenko, E.N., Alchinova, I.B., Karganov, M.Y., Skalny, A.V., Nikonorov, A.A. (2016). Decreased adipose tissue zinc content is associated with metabolic parameters in high fat fed Wistar rats. Acta Sci. Pol. Technol. Aliment. 15, 99-105. doi: 10.17306/J.AFS.2016.1.10.

Vidé, J., Bonafos, B., Fouret, G., Benlebna, M., Poupon, J., Jover, B., Casas, F., Jouy, N., Feillet-Coudray, C., Gaillet, S., Coudray, C. (2018). *Spirulina platensis* and silicon-enriched spirulina equally improve glucose tolerance and decrease the enzymatic activity of hepatic NADPH oxidase in obesogenic diet-fed rats. Food Funct. 9, 6165-6178. doi: 10.1039/c8fo02037j

Virto, L., Cano, P., Jiménez-Ortega, V., Fernández-Mateos, P., González, J., Esquifino, A.I., Sanz, M. (2018). Obesity and periodontitis: An experimental study to evaluate periodontal and systemic effects of comorbidity. J. Periodontol. 89, 176-185. doi: 10.1902/jop.2017.170355.

Wei, Y., Hong, Y., Hou, P., Xu, X. (2019). Effect of sugar-free Qishan granules on glucose and lipid metabolism and insulin resistance in a rat model of prediabetes. J. Tradit. Chin. Med. 39, 535-541.

Wen, J.J., Gao, H., Hu, J.L., Nie, Q.X., Chen, H.H., Xiong, T., Nie, S.P., Xie, M.Y. (2019). Polysaccharides from fermented *Momordica charantia* ameliorate obesity in high-fat induced obese rats. Food Funct. 10, 448-457. doi: 10.1039/c8fo01609g

Wong, S.K., Chin, K.Y., Suhaimi, F.H., Ahmad, F., Ima-Nirwana, S. (2018). Exploring the potential of tocotrienol from *Bixa orellana* as a single agent targeting metabolic syndrome and bone loss. Bone. 116, 8-21. doi: 10.1016/j.bone.2018.07.003.

Wu, Y.Y., Zha, Y., Liu, J., Wang, F., Xu, J., Chen, Z.P., Ding, H.Y., Sheng, L., Han, X.J. (2016). Effect of berberine on the ratio of high-molecular weight adiponectin to total adiponectin and adiponectin receptors expressions in high-fat diet fed rats. Chin. J. Integr. Med. doi: 10.1007/s11655-016-2518-x.

Zayed, E.A., AinShoka, A.A., El Shazly, K.A., Abd El Latif, H.A. (2018). Improvement of insulin resistance via increase of GLUT4 and PPARγ in metabolic syndrome-induced rats treated with omega-3 fatty acid or l-carnitine. J. Biochem. Mol. Toxicol. 32, e22218. doi: 10.1002/jbt.22218.

Zhang, J., Wang, O., Guo, Y., Wang, T., Wang, S., Li, G., Ji, B., Deng, Q. (2016). Effect of increasing doses of linoleic and α-linolenic acids on high-fructose and high-fat diet induced metabolic syndrome in rats. J. Agric. Food. Chem. 64, 762-772. doi: 10.1021/acs.jafc.5b04715.

Zhang, J., Zhao, L., Cheng, Q., Ji, B., Yang, M., Sanidad, K.Z., Wang, C., Zhou, F. (2018). Structurally different flavonoid subclasses attenuate high-fat and high-fructose diet induced metabolic syndrome in rats. J. Agric. Food. Chem. 66, 12412-12420. doi: 10.1021/acs.jafc.8b03574

Zhao, L., Zhang, Q., Ma, W., Tian, F., Shen, H., Zhou, M. (2017). A combination of quercetin and resveratrol reduces obesity in high-fat diet-fed rats by modulation of gut microbiota. Food Funct. 8, 4644-4656. doi: 10.1039/c7fo01383c.
